# Predator–Prey Behavior of Droplets Propelling Through Self‐Generated Channels in Crystalline Surfactant Layers

**DOI:** 10.1002/anie.202502352

**Published:** 2025-04-15

**Authors:** Priyanshu Singh, Peter A. Korevaar

**Affiliations:** ^1^ Institute for Molecules and Materials Radboud University Heyendaalseweg 135 Nijmegen 6525 AJ The Netherlands

**Keywords:** Active droplets, Marangoni effect, Motility, Out‐of‐equilibrium, Systems chemistry

## Abstract

Motile droplets provide an attractive platform for liquid matter‐based applications and protocell analogues displaying life‐like features. The functionality of collectively operating droplets increases by the advance of well‐designed (physico)chemical systems directing droplet–droplet interactions. Here, we report a strategy based on crystalline surfactant layers at air/water interfaces, which sustain the propulsion of floating droplets and at the same time shape the paths for other droplets attracted by them. First, we show how decylamine forms a closed, crystalline layer that remains at the air/water interface. Second, we demonstrate how aldehyde‐based oil droplets react to decylamine in the crystalline layer by forming an imine, causing the droplets to move through the layer while leaving behind an open channel (comparable to “Pac‐Man”). Third, we introduce tri(ethylene glycol) monododecylether (C_12_E_3_) droplets in the crystalline layer. The crystalline layer suppresses the motion of the C_12_E_3_ droplets, however, the aldehyde droplets create surface tension gradients upon depletion of surfactants from the air/water interface, thereby driving Marangoni flows that attract the C_12_E_3_ droplets as well as the myelin filaments they grow: Causing the C_12_E_3_ droplets to chase, and ultimately catch, the aldehyde droplets along the channels they have created, featuring a predator‐prey analogy established at an air/water interface.

## Graphical abstract

A crystalline layer is formed by an amphiphilic decylamine surfactant at the air/water interface. Aldehyde‐containing ester oil droplets self‐propel by consuming amines from the crystalline layer, thereby generating open channels along which myelin‐forming amphiphile droplets chase and ultimately catch the aldehyde droplets in a predator–prey analogy.

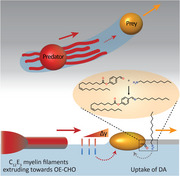

## Introduction

Droplets provide an attractive platform to establish spontaneous emergence of life‐like behavior from synthetic building blocks. The versatility of droplets –often oil‐based surrounded by aqueous media– allows their content to be easily adjusted to establish controllable interactions with “the outside world” – typically the aqueous solution or other droplets. Well‐designed systems of chemical reactions and physicochemical interactions allow droplets to become motile and display features in analogy to microorganisms, ranging from collective swarming^[^ ^1^, ^2^, ^3^, ^4^, ^5^, ^6^ ^]^ *quorum* sensing^[^ ^7^ ^]^ and chemo‐ or phototaxis,^[^ ^8^, ^9^, ^10^, ^11^, ^12^, ^13^, ^14^, ^15^ ^]^ or to serve as protocell analogues in origins‐of‐life studies.^[^ ^16^, ^17^, ^18^ ^]^ At the same time, droplet‐based systems provide functional applications as diverse as multiphase reactors,^[^ ^19^, ^20^ ^]^ reconfigurable structures,^[^ ^21^, ^22^, ^23^ ^]^ maze solving,^[^ ^24^ ^]^ modifiable liquid optics,^[^ ^25^ ^]^ controlled surface patterning,^[^ ^26^, ^27^ ^]^ new modes of sensing,^[^ ^28^, ^29^ ^]^ and unconventional computing.^[^ ^30^, ^31^ ^]^

A common feature of motile droplet systems is that they operate out‐of‐equilibrium. By establishing different droplet types in source–sink configurations, concentration gradients can be sustained amongst them, which in turn direct the motility of the droplets in nonreciprocal configurations. For example, Zarzar and coworkers exploited bromo‐octane and fluorinated oil droplets immersed in aqueous micellar solutions, which display a difference in micellar solubilization rate.^[^ ^32^, ^33^ ^]^ Hence, a concentration gradient of filled and empty micelles surrounds the droplets, causing unequal adsorption of surfactant molecules at their front and rear sides, such that they start to chase each other through the solution in analogy to predator–prey behavior. Recently, our research group developed a source–sink system that self‐organizes into 2D patterns at air/water (a/w) interfaces. Surface tension gradients can readily be established along a/w interfaces, driving Marangoni flows that in turn direct the motility and interactions of active droplets at mesoscopic or macroscopic length scale.^[^ ^24^, ^34^, ^35^, ^36^, ^37^, ^38^, ^39^, ^40^, ^41^ ^]^ Combining droplets that release surfactants (source) and oil‐based drain droplets (sink) that in turn deplete these surfactants from the a/w interface allowed us to sustain surface tension gradients that drive Marangoni flows along the a/w interface. The concomitant assembly of the surfactants (tri‐ and tetra(ethyleneglycol) monododecyl ether, C_12_E_3_ and C_12_E_4_) into multilamellar filaments (myelins) that grow from the source droplet get directed by these Marangoni flows toward the drain.^[^ ^42^, ^43^, ^44^ ^]^ Together, the motion of source and drain droplets with respect to each other relies on the balance between Marangoni flows and elastocapillary forces due to the adhesion of myelins to the drain droplets.

**Figure 1:**
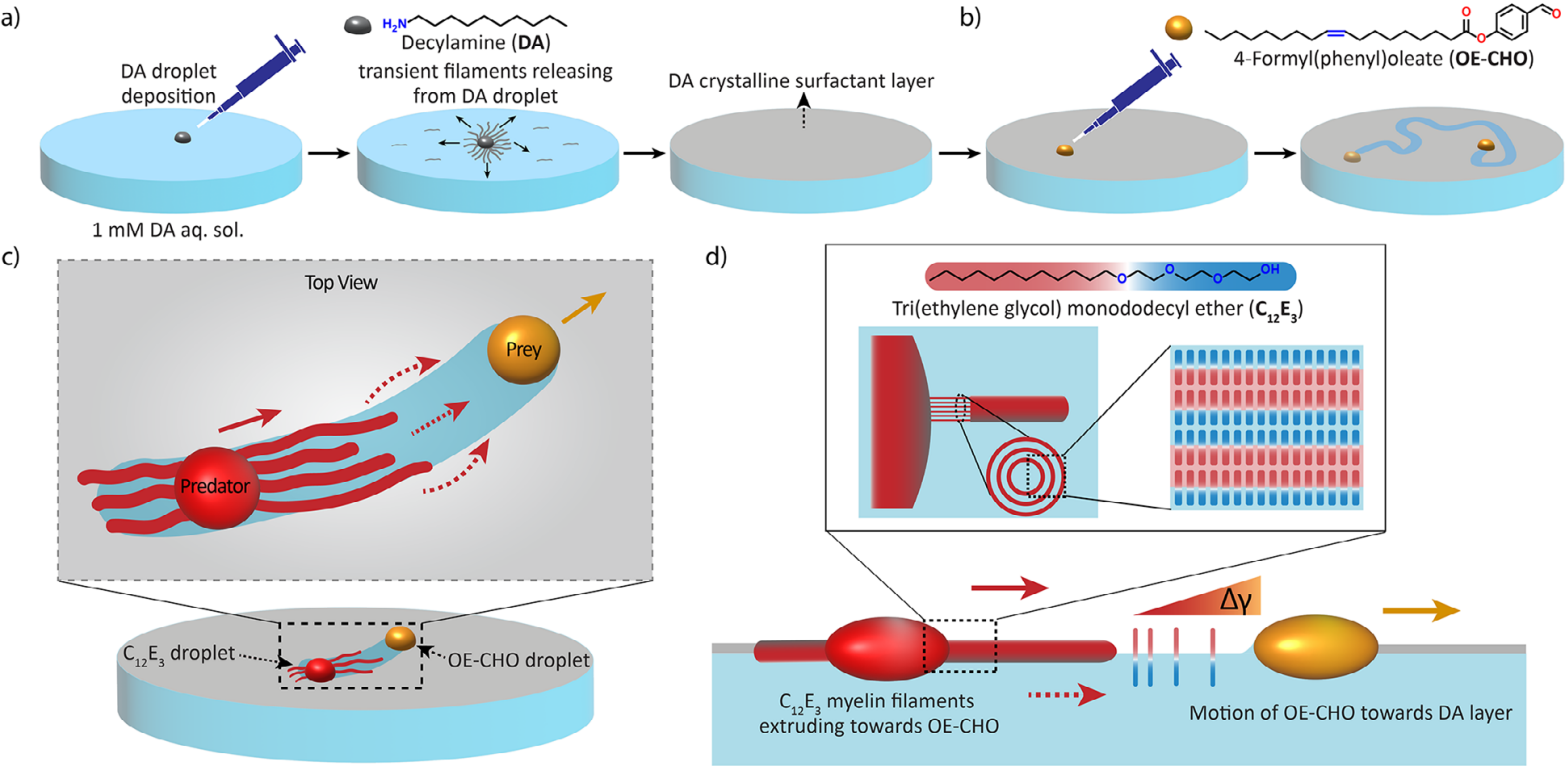
Predator–prey behavior of floating droplets self‐propelling through a decylamine surfactant layer. a) Schematic representation of the surfactant decylamine (DA) droplet deposition at the air/water interface, forming a crystalline layer. b) We introduce an ester‐based oil droplet 4‐formyl(phenyl)oleate (OE‐CHO), containing an aldehyde functional group. Upon depositing onto the DA layer, the OE‐CHO droplet shows a self‐propelled motion and creates a channel through the DA layer. c) Predator–prey interaction amongst a C_12_E_3_ droplet (predator) and an OE‐CHO droplet (prey) moving along a self‐created channel through the DA layer. d) Myelin filaments grow from a C_12_E_3_ droplet (red) floating on an aqueous solution. The motile OE‐CHO droplet (yellow) absorbs the C_12_E_3_ surfactants, generating a surface tension gradient Δγ at the a/w interface along the channel, which attracts the C_12_E_3_ predator toward the OE‐CHO prey.

Whereas such surface tension‐related phenomena amongst floating droplets typically occur at “naked” a/w interfaces, we report a strategy to use crystalline surfactant layers at a/w interfaces to direct these surface tension gradients. Our crystalline layers “fuel” the propulsion of motile prey droplets and at the same time shape the paths for predator droplets chasing the prey. Departing from droplets that move along surfactant‐loaded solid substrates by consuming the surfactant,^[^ ^26^, ^45^ ^]^ we reasoned that consumption of surfactants deposited as a solid layer on top of an aqueous solution creates paths that in turn direct the source–sink interactions. Indeed, we show how oil droplets moving through the crystalline surfactant layer create channels, and concomitantly serve as drains that attract myelins: establishing a unique predator–prey system where the predator chases the prey along the channels that have been created by the prey. First, we establish a crystalline surfactant layer formed by decylamine (DA) at the air–water interface (Figure 1a). Next, we introduce an ester oil droplet containing an aldehyde moiety (OE‐CHO, 4‐formyl(phenyl)oleate), which is prone to react with the amine of DA, allowing the droplet to create a channel as it moves through the crystalline layer along self‐avoiding paths (Figure 1b). Finally, we demonstrate and rationalize the predator–prey behavior of OE‐CHO, as it attracts as a drain droplet a C_12_E_3_ droplet, which in turn chases as a predator the OE‐CHO prey along the channels (Figure 1c,d).

## Results and Discussion

### Formation of Decylamine‐Based Crystalline Surfactant Layer at Air/Water Interfaces

**Figure 2:**
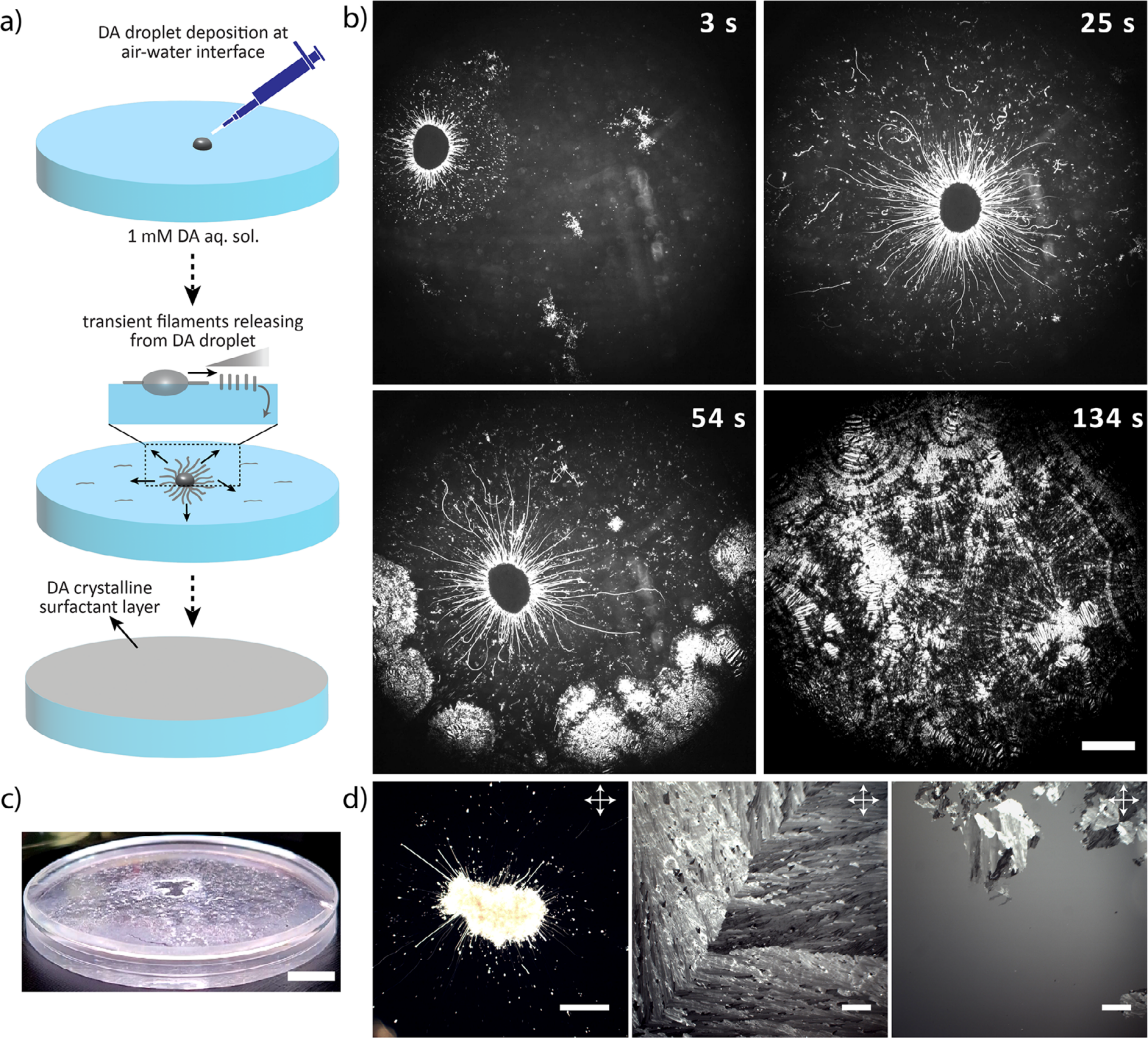
Decylamine (DA) crystalline layer formation at the air/water interface. a) Schematic representation of DA microdroplet deposition at the a/w interface, releasing short transient filaments which are transferred away from the droplet by outbound Marangoni flows and contribute to the build‐up of the crystalline layer at the a/w interface. b) Optical microscopy recording of a 0.5 µL DA droplet deposited at the a/w interface, featuring the formation of short transient filaments and the subsequent growth of the solid surfactant layer. The scale bar represents 2 mm. c) Photograph, acquired under an angle from the side, featuring the white solid DA layer covering the entire surface of the aqueous solution in a Petri dish. The scale bar represents 15 mm. d) Polarized optical microscopy images of a 0.5 µL DA droplet (left); the DA layer with crystalline nature (middle) and an open spot in a damaged DA layer, featuring the naked a/w interface (right). The scale bars represent 2 mm.

We use the hydrophobic surfactant molecule decylamine (DA) to establish a crystalline surfactant layer as a medium for the motile droplets. As shown in Figure 2a,b, and Video S1, when a droplet of DA (0.5 µL) is deposited at the air/water (a/w) interface of a Petri dish with an aqueous DA solution (1 mM, 6 mL), it starts to grow small filamentous structures and over time, the aqueous solution is covered by a thin crystalline layer. The extrusion of the filaments from the DA droplet can be observed in optical microscopy (Figure 2b), and holds a similarity to the growth of the myelins from C_12_E_3_‐based droplets.^[^ ^42^ ^]^ Indeed, DA lowers the surface tension of the a/w interface to approx. 24 mN m^−1^, as confirmed by surface tension measurements (Figure S1), and thereby drives outbound Marangoni flows from the DA droplet. At the end of the DA‐based filaments, which reach a length of approx. 2–3 mm, short pieces detach and get carried away by the outbound Marangoni flow toward the walls of the Petri dish (diameter 35 mm). As a result, the DA layer grows from the periphery of the a/w interface, and gets filled up as more DA material is transferred by the outbound Marangoni flow to the edge of the layer, which reaches the DA droplet over a time course of approx. 1 min, resulting in a fully covered a/w interface. To the best of our knowledge, the Marangoni‐driven formation of the DA layer at the a/w interface has not been reported before.

The formation of the DA crystalline layer is observable to the naked eye, resembling a white snowlike appearance at the interface (Figure 2c). The birefringent appearance in polarized optical microscopy confirms the (liquid) crystalline nature of the DA layer (Figure 2d). DA crystalline layers that have been grown on a 1 mM DA solution remain stable over a time period of approx. 60 min (Figure S2), after which cracks appear in the layer. Importantly, inclusion of DA (1 mM) in the aqueous solution enhances the stability of the layer, presumably by counteracting the depletion of DA from the solid layer to the underlying aqueous solution. Furthermore, the DA layer stability is sensitive to the pH of the aqueous solution, which is approx. 9.3 for a 1 mM DA solution: With aqueous solutions of 5 mM HCl and 5 mM NaOH, DA rapidly dissolves or forms phase separated floating droplets, respectively (Figures S3, S4).

### Self‐Propelled Motion of Oil Droplets Through the DA Crystalline Layer

**Figure 3:**
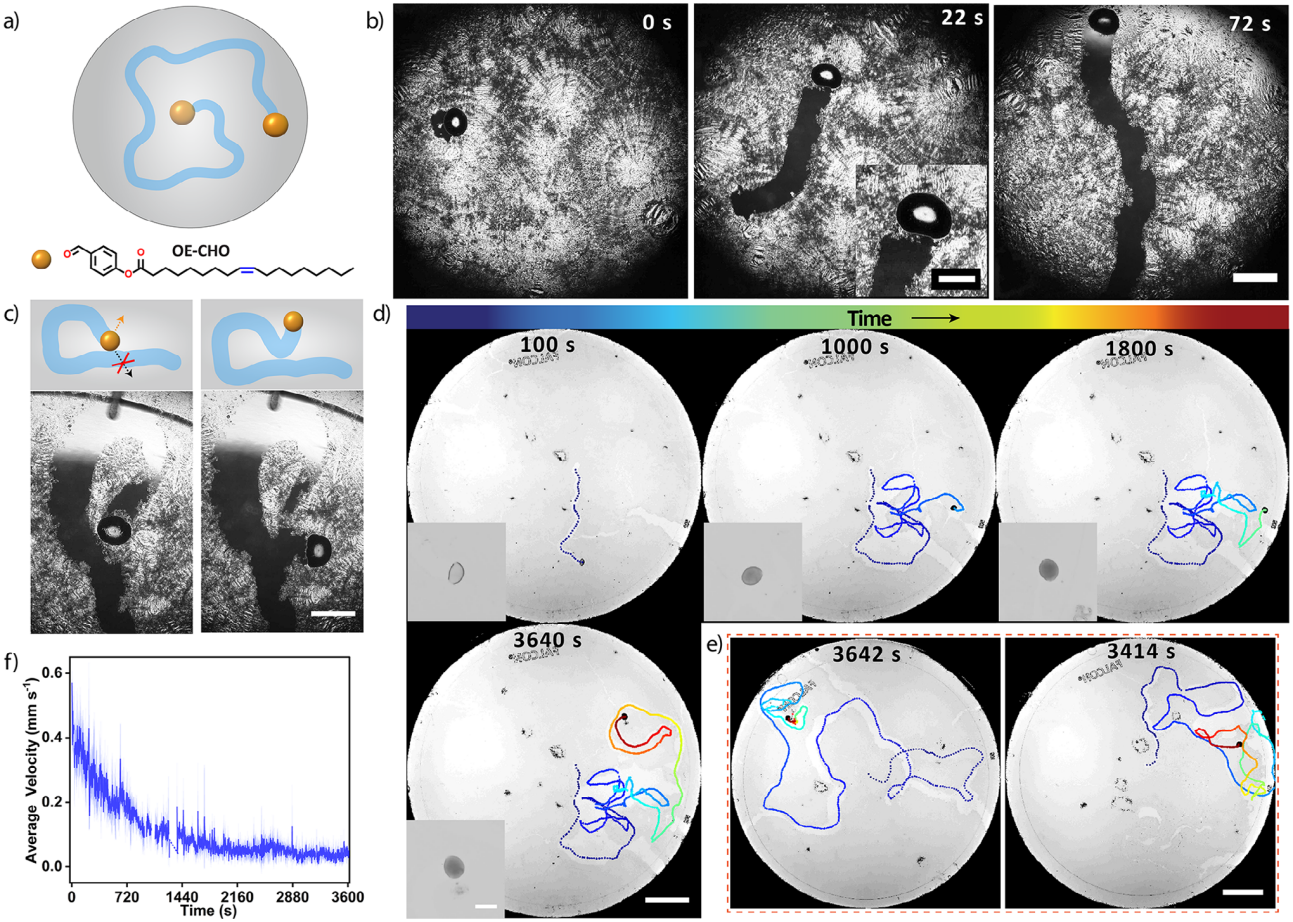
Self‐propelled motion of OE‐CHO oil droplet through the DA crystalline layer. a) Schematic representation of the self‐propelled motion of a OE‐CHO oil droplet, creating an open channel in the DA layer. b) Optical microscopy recording of a 0.5 µL OE‐CHO oil droplet self‐propelling through a DA crystalline layer by creating an open channel at the a/w interface. During the self‐propelled motion, the shape of the droplet changes from spherical to oblate. The scale bars represent 2 mm and 0.5 mm (inset), respectively. c) The OE‐CHO droplet shows self‐evading behavior by avoiding the open channel formed earlier in the DA crystalline layer by changing the direction of motion. The scale bar represents 2 mm. d) Time‐dependent positioning traces acquired on camera recordings of an OE‐CHO droplet (1.0 µL) deposited on a DA crystalline layer (1 mM DA, 60 mL in 100 mm diameter Petri dish). Self‐propulsion of the droplet was sustained for more than 3600 s, with a gradual decrease in velocity over time. The scale bars represent 15 and 2 mm (inset), respectively. e) Time‐dependent positioning traces acquired on camera recordings of two other experiments in analogy to the experiment shown in d). The scale bar represents 15 mm. The camera recordings were contrast enhanced to visualize the channels formed in the DA crystalline layer (Supporting Information). f) Velocity of the OE‐CHO droplet versus time, averaged over the three replicate experiments shown in d) and e).

We used the oleic ester derivative OE‐CHO (4‐formyl(phenyl)oleate) as oil‐based compound for the motile droplets to move through the DA layer. OE‐CHO was synthesized via an esterification of 4‐hydroxybenzaldehyde with oleic acid, following a modified procedure of Rostoll–Berenguer et al^[^ ^46^ ^]^ (Supporting Information). Indeed, when depositing a 0.5 µL droplet of OE‐CHO onto a DA layer that has been formed at the a/w interface, the droplet starts dissolving the DA layer and creating a millimeter‐wide channel as it self‐propels through the DA layer (Figure 3a,b, Video S2 and Figure S5). Concomitantly, the initial spherical shape of the oil droplet changes to an oblate‐like shape during the self‐propulsion. Even though the motion of the droplet is random, it evades the path that it has created through the DA layer – corroborating the role of DA as a driving force for the propulsion (Figure 3c). We note that comparable self‐avoiding tracks were observed in the motion of mm‐sized condensate water droplets at solid interfaces, driven by the interfacial energy release upon merging with newly condensed µm‐sized droplets.^[^ ^47^ ^]^

To enhance the lifetime of the droplets, typically 5 to 12 min before their collapse to the wall of the Petri dish, we continued our experiments with larger Petri dishes. When depositing a 1.0 µL OE‐CHO droplet at the DA layer that has been formed in a large area Petri dish (100 x 15 mm, 60 mL of 1 mM DA), oil droplets were observed to sustain their motion for up to 60 min (Figure 3d,e, Video S3). We note that when no DA layer is present, the OE‐CHO droplet – when deposited either on MQ water or a 1 mM DA solution – rapidly bounces to the wall of Petri dish upon deposition (Figure S6). In the DA layer, the droplets move with an initial velocity of approx. 0.4 mm s^−1^, and gradually slow down over a timecourse of 30 min to 0.05 mm s^−1^ (Figures 3f, S7). In the camera recordings, the droplets acquire an amorphous appearance which we ascribe to the acquisition of DA by the OE‐CHO droplet (Figure 3d). With four droplets being deposited at a 100 mm diameter DA layer, we observe both droplets merging or colliding to the wall as factors limiting their lifetime (Figure S8, Video S4).

### Mechanism of Self‐Sustained OE‐CHO Oil Droplet Motion

**Figure 4:**
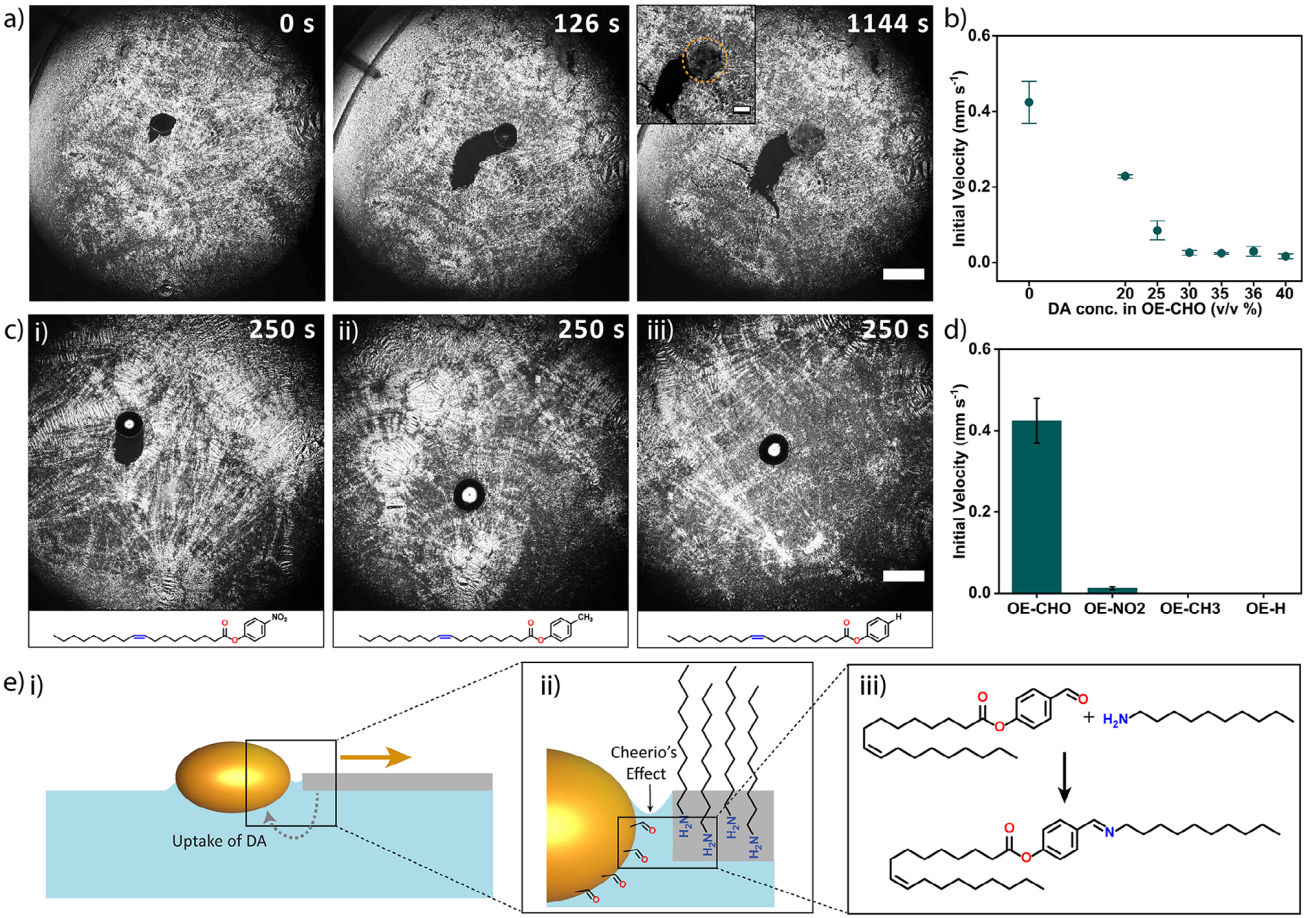
The propulsion mechanism of OE‐CHO droplets through the DA crystalline layer. a) Optical microscopy recording of an OE‐CHO droplet containing 40 v/v% DA (0.5 µL), which suppresses its motility through the DA crystalline layer. Over time the shape of the oil droplet changes and it gets a crystalline appearance (similar to the DA crystalline layer, as exemplified at *t* = 1144 s). The scale bars represent 2 and 0.5 mm (inset), respectively. b) Graph of the initial velocity of OE‐CHO‐based droplets (acquired over first 18 s after deposition) through a DA crystalline layer versus DA content of the droplets (*n* = 3 experiments per data point). c) Optical microscopy recordings of OE‐NO_2_, OE‐CH_3_, and OE‐H droplets (0.5 µL) deposited on a DA crystalline layer. The scale bar represents 2 mm. d) Bar diagram of the initial velocity of the oleate ester derivatives (acquired over first 18 s after deposition, *n* = 3 experiments per compound). e) Schematic representation (side view) of the propulsion of an OE‐CHO droplet (yellow) through a DA crystalline layer (grey) on an aqueous solution (blue). Upon formation of an imine, the OE‐CHO droplet takes up DA from the layer. The capillary attraction, due to the overlap in the air/water menisci (the “Cheerio's” effect) keeps the OE‐CHO droplet in contact with the DA layer, causing it to create a channel as it moves through the DA crystalline layer.

We hypothesized that the motility of the OE‐CHO droplet, and its decline over time, is related to the uptake of DA. To investigate the effect of DA on the motility of the droplet, we mixed DA with OE‐CHO in different ratios, varying from 20 to 40 v/v% DA in OE‐CHO (Figures S9–S13). As shown in Figure 4a,b, inclusion of DA declines the initial velocity of the droplet, and droplets with a DA content ≥ 30 v/v% show hardly any self‐propulsion through the DA crystalline layer. Importantly, the droplet velocity is relatively independent of the concentration of DA in the aqueous solution (Figure S14). Next, we speculate that the uptake of DA by the droplet is associated with the aldehyde moiety of OE‐CHO, exposed at the surface of the droplet, which reacts with the primary amine of the DA crystalline layer by forming a dynamic covalent imine bond (Schiff base). Indeed, the imine formation is confirmed by ^1^H‐NMR spectroscopy analysis performed on an OE‐CHO droplet that has been in contact with a DA crystalline layer for 30 min (Figure S15). The formation of the nonpolar imine, which is preferentially solvated by the hydrophobic interior of the OE‐CHO droplet, will enhance the incorporation of the hydrophilic ─NH_2_ head group of DA by the droplet. Gratifyingly, upon substituting the aldehyde group at the para position of the phenyl ring for either an electron withdrawing nitro group (OE‐NO_2_), an electron donating methyl group (OE‐CH_3_), or a hydrogen (OE‐H), the motility of the droplet through the DA layer was observed to decline substantially – corroborating the role of imine formation in the OE‐CHO droplet self‐propulsion (Figure 4c,d).

The self‐propulsion of OE‐CHO upon forming a channel through the DA layer indicates that the DA uptake breaks the symmetry of the droplet – as confirmed by its oblate‐like shape. An imbalance of the forces exerted on the droplet can be explained by the “Cheerio's” effect,^[^ ^48^ ^]^ which involves the capillary attraction emerging from the overlap in the air/water menisci of the OE‐CHO droplet and the DA crystalline layer, both floating on water. As the DA crystalline layer gets dissolved into the droplet, the capillary attraction will cause the droplet to keep approaching the continuously moving edge of the DA layer (Figure 4e). Together, the dissolution of DA molecules inside the OE‐CHO oil phase upon the formation of an imine leads to the transformation of chemical energy into kinetic energy as the droplet moves through the DA crystalline layer.

### C_12_E_3_‐Based Myelin Growth in the DA Crystalline Layer

**Figure 5:**
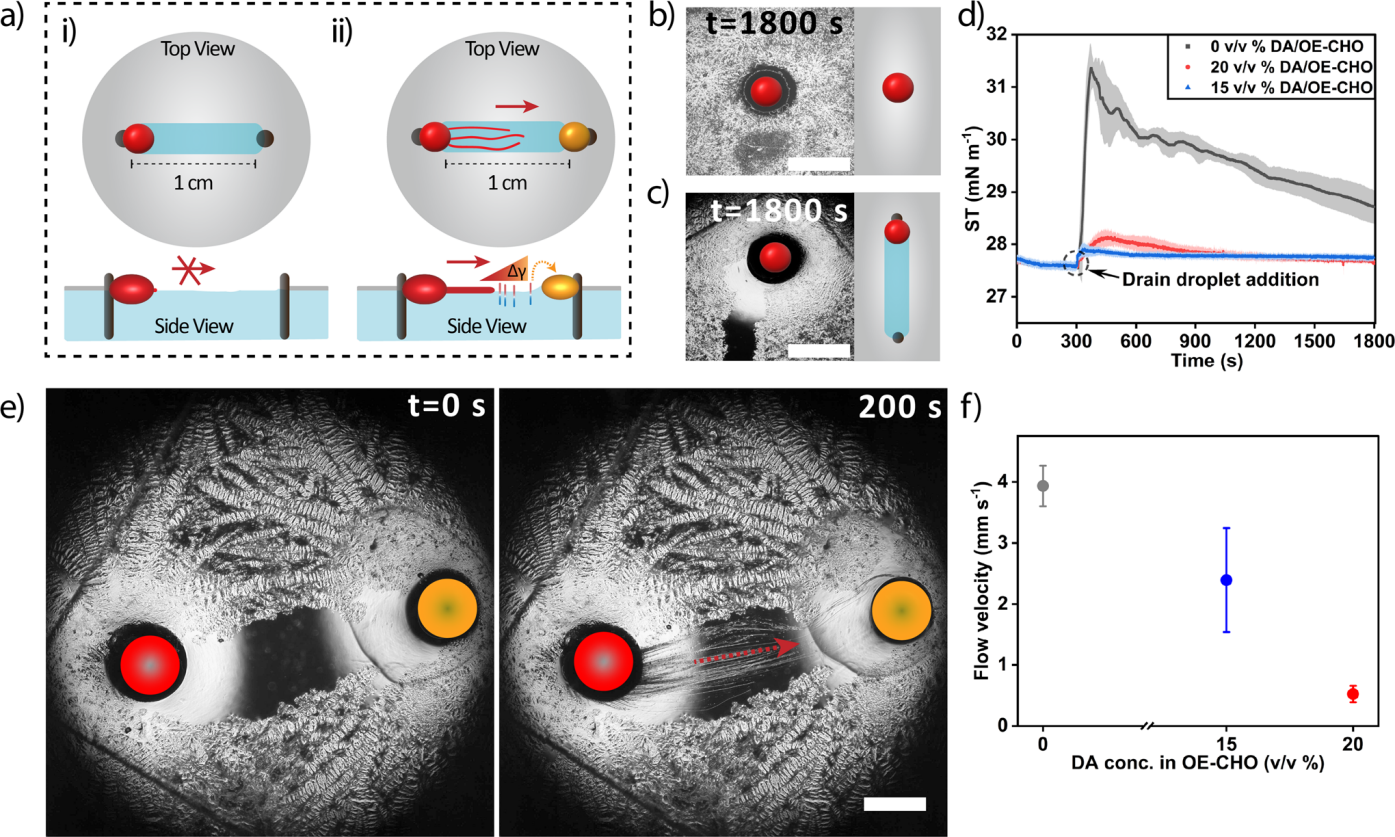
Attraction of myelins by OE‐CHO drain droplet. a) Schematic representation of open channels in the DA crystalline layer. The myelin filament growth is inhibited in the absence of the OE‐CHO oil droplet (i); in the presence of the OE‐CHO oil droplet, the C_12_E_3_ myelins grow and are directed toward the OE‐CHO droplet (ii). b) Optical microscopy image of a 1.0 µL C_12_E_3_ droplet deposited in a DA solid surfactant layer, featuring no myelin growth after 1800 s. c) A C_12_E_3_ droplet (1.0 µL) placed in an open channel amongst the DA crystalline layer does not feature myelin growth at *t* = 1800 s. The scale bars represent 2 mm. d) Surface tension (ST) versus time, acquired on a 0.5 mM C_12_E_3_ solution. At *t* = 300 s (arrow), a 0.5 µL OE‐CHO droplet (0; 15; and 20 v/v% DA) is deposited at the a/w interface, and the increase in ST indicates the drain effect of the OE‐CHO droplet. Each curve is averaged over *n* = 3 separate experiments. e) Optical microscopy recordings of myelins growing from a 1.0 µL C_12_E_3_ source droplet (red), directed along a 1 cm‐long open channel created in the DA crystalline layer toward a 0.5 µL OE‐CHO drain droplet positioned at the opposite side of the channel (yellow). The scale bar represents 2 mm. f) Flow velocity, probed via free‐floating particles (*n* = 3 per experiment) in the open‐channel experiments with different DA content in the OE‐CHO droplets.

Next, we explore the behavior of C_12_E_3_ droplets in the presence of the DA crystalline layer at the air–water interface. Earlier, our group showed that a C_12_E_3_ droplet, when deposited at an air–water interface, takes up water from the surrounding medium and forms filaments known as myelins, as shown in Figure 1d. This process is driven by an outbound Marangoni flow, which promotes the extrusion of these myelin filaments from the C_12_E_3_ droplet along the a/w interface. Here, we deposited a 1.0 µL C_12_E_3_ droplet onto an aqueous solution with a DA crystalline layer. As shown in Figure 5b, the C_12_E_3_ droplet adheres to the DA layer and does not show any myelin growth over a time course of 1800 s. This inhibition of myelin formation can be attributed to the differences in surface tension: the surface tension of an aqueous solution with C_12_E_3_ is approx. 27.5 mN m^−1^ (Figure 5d), whereas in the presence of 1 mM DA the surface tension can be suppressed to approx. 24 mN m^−1^ (Figure S1). Thereby, the surface active DA restricts the outbound Marangoni flow that would be initiated upon C_12_E_3_ release, thus suppressing the myelin growth.

To probe the interaction of C_12_E_3_ and OE‐CHO droplets along channels, we formed a 1 cm open channel within the DA crystalline layer. A C_12_E_3_ droplet was deposited at one end of the channel, and kept in position by tethering it to the meniscus of a metal pin, using the “Cheerio's” effect (Figures 5a, S16). Despite the open structure of the channel, no myelin growth was observed from the C_12_E_3_ droplet along the channel (Figure 5c). This lack of myelin formation suggests that, in the presence of the DA layer, the droplet does not initiate any C_12_E_3_‐driven Marangoni flow capable of overcoming the stronger surface activity of DA at the a/w interface. However, when a OE‐CHO droplet is positioned at the opposite side of the channel, myelins emerge from the C_12_E_3_ droplet and get attracted toward the OE‐CHO droplet (Figure 5e, Video S5). This observation indicates that the OE‐CHO droplets can also function as a drain for C_12_E_3_ and DA surfactants and thereby generate a surface tension gradient required for myelin attraction. Indeed, surface tension measurements show an increase in surface tension upon deposition of an OE‐CHO droplet on a 0.5 mM C_12_E_3_ solution (without DA, Figure 5d). Also, in the absence of C_12_E_3_, we observe a Marangoni flow toward the OE‐CHO droplet, presumably due to depletion of DA (Figure S6). We note that OE‐CHO by itself has limited surface tension activity in comparison to C_12_E_3_ and DA (Figure S17). Interestingly, the DA crystalline layer can be regenerated when formed on a 2.5 mM DA solution, as demonstrated by channels formed by an OE‐CHO droplet that close again over a time course of approx. 13 min (Figure S18). However, such concentrations suppress C_12_E_3_ myelin growth due to the strong surface activity of DA. In fact, to avoid the OE‐CHO‐induced surface tension gradients being suppressed by DA surfactants, in the predator‐prey experiments (vide infra) we have reduced the DA concentration in the aqueous medium to 0.5 mM.

### Predator–Prey Like Behavior by C_12_E_3_ and OE‐CHO Droplets

**Figure 6:**
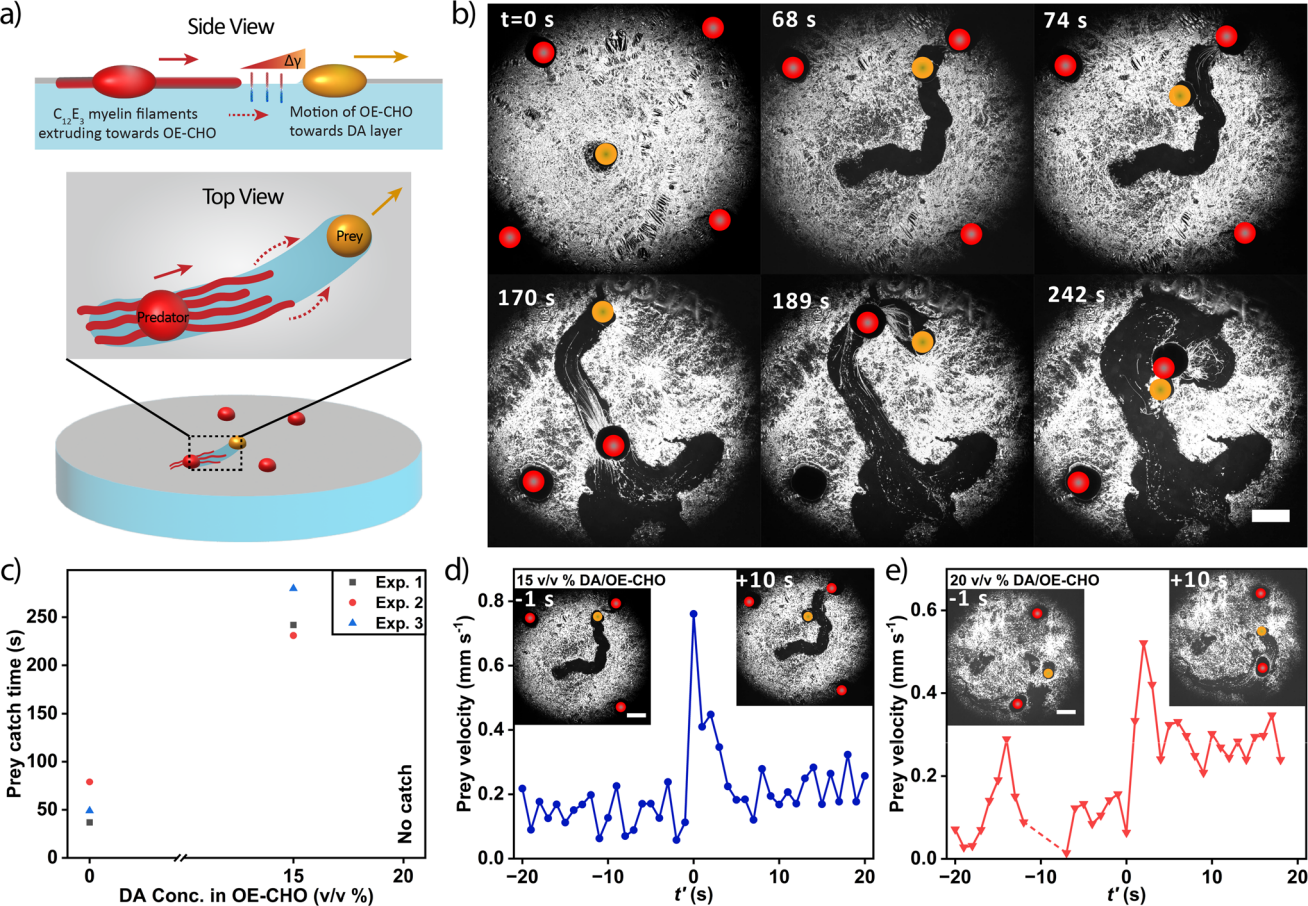
Predator–prey interaction amongst C_12_E_3_ and OE‐CHO droplets. a) Schematic representation of predator–prey interaction between C_12_E_3_ (predator, red) and OE‐CHO (prey, yellow) droplets, deposited on a DA crystalline layer (grey). b) Optical microscopy recordings showing the predator–prey interaction of four C_12_E_3_ droplets (0.5 µL, red) and one prey droplet (15 v/v% DA in OE‐CHO, 0.5 µL, yellow), deposited on a DA crystalline layer formed on a 0.5 mM DA aqueous solution in a 35 mm Petri dish at *t* = 0 s. The C_12_E_3_ droplet started chasing at *t* = 74 s and later caught the OE‐CHO oil droplet at *t* = 242 s. c) Prey catch time versus DA content in OE‐CHO prey droplets, observed in predator–prey experiments as schematically shown in a). d), e) Time‐dependent velocity of an OE‐CHO prey droplet (d, 15 v/v% DA; e, 20 v/v% DA), prior to (i.e., *t’* < 0 s) and while being chased (*t’* ≥ 0 s) by a C_12_E_3_ droplet, as shown by the optical microscopy recordings (insets). The scale bars represent 2 mm.

We explored a unique interaction amongst C_12_E_3_ and OE‐CHO droplets and the DA crystalline layer, exhibiting characteristics that are analogous to predator–prey behavior. In this system, C_12_E_3_ droplets act as “predators”, while OE‐CHO droplets function as “prey”, as schematically shown in Figure 6a. Upon deposition of four C_12_E_3_ droplets onto the DA crystalline layer that has been formed at the air/water interface, the C_12_E_3_ droplets become immobilized, with initial inhibition of myelin formation (Figure 6b, Video S6). However, introduction of an OE‐CHO oil droplet amongst the C_12_E_3_ droplets initiates a dynamic response: The OE‐CHO droplet self‐propels across the DA layer and creates an open channel near the C_12_E_3_ droplet. As the self‐propelling OE‐CHO droplet approaches the immobilized C_12_E_3_ droplet, it triggers myelin growth from the C_12_E_3_ source. These myelins extend, via the open channel that has been created, in the direction of the OE‐CHO droplet, appearing to “chase” its path as it moves along the DA layer. This responsive myelin formation is driven by a reactivation of local Marangoni flow as the C_12_E_3_ droplet responds to the proximity of the OE‐CHO droplet. The configuration of four C_12_E_3_ predator droplets around one OE‐CHO prey droplet enhances the chance of one predator catching the prey, on average within 55 ± 22 s (*n* = 3 experiments, Video S6, Figure 6c). If only one C_12_E_3_ predator droplet is present, the prey is more likely to bounce to the wall of the Petri dish prior to being caught by the predator (Figure S19); in the absence of a DA crystalline layer, the OE‐CHO and C_12_E_3_ droplets merge upon deposition at the a/w interface within a few seconds (Figure S20).

Modifying the droplet composition allows to tune the dynamics of the predator–prey interactions. Introducing DA in the OE‐CHO droplet slows down the velocity of the droplet progressing through the DA crystalline layer (Figure 4b). We reasoned that a more sustained predator–prey behavior requires a slower attraction of the C_12_E_3_ droplet toward the OE‐CHO droplet. Importantly, inclusion of DA in the OE‐CHO droplet suppresses its attraction of myelins, as shown in open‐channel experiments with 15 and 20 v/v% DA included in the OE‐CHO droplet, respectively (Figure 5f). This reduced attraction can be ascribed to a suppressed depletion of C_12_E_3_ from the a/w interface, as observed in surface tension measurements with 15 and 20 v/v% DA droplet deposited on a 0.5 mM C_12_E_3_ solution (Figure 5d). The further decline of drain activity when increasing the DA content in the OE‐CHO droplet from 15 to 20 v/v% is ascribed to a reduced depletion of DA from the a/w interface by a 20 v/v% DA droplet. Next, when depositing a OE‐CHO prey droplet with 15 v/v% DA amongst four C_12_E_3_ predator droplets, we observed the catch time to increase to 251 ± 26 s (*n* = 3), whereas OE‐CHO droplets with 20 v/v% DA either directly bounced into a predator, or escaped from the predator droplets (*n* = 3, Video S6, Figure 6c). Finally, the predator–prey characteristics of our system are corroborated by experimental observations where an OE‐CHO droplet closely approaches a DA layer‐embedded C_12_E_3_ droplet, followed by a sudden rupture of the DA crystalline layer at *t’* = 0 s that allows the C_12_E_3_ droplet to chase the OE‐CHO droplet (15 v/v% DA, Figure 6d). Under such conditions, the velocity of the OE‐CHO droplet is observed to suddenly increase at *t’* = 0. In analogy, Figure 6e showcases a 20 v/v% DA in OE‐CHO droplet that increases its velocity when the DA layer separating it from a neighboring C_12_E_3_ droplet disintegrates at *t’* = 0 s – showcasing an apparent “fleeing” from the predator which can be ascribed to the Marangoni flow from the C_12_E_3_ to the OE‐CHO droplet (Figure 5e), pushing forward the prey droplet.

## Conclusion

We demonstrate predator–prey behavior of surface‐active myelin‐growing amphiphile droplets and aldehyde‐based oil droplets on an amine‐based crystalline layer formed at the air/water interface. First, we show how outbound Marangoni flows from a decylamine (DA) droplet allow to form a closed, crystalline layer that remains at the a/w interface. Next, we exploited an oleate ester derivative containing an aldehyde OE‐CHO, which reacts to the amine of the crystalline layer, resulting in imine formation and driving the propulsion of OE‐CHO microdroplets through the layer. While moving, OE‐CHO droplets consume the DA crystalline layer in analogy to the “Pac‐Man” game, creating millimeter‐wide open channels. Thereafter, we introduce a C_12_E_3_ droplet on the DA crystalline layer. The DA layer suppresses the myelin growth, however, OE‐CHO droplets can create surface tension gradients upon depletion of surfactants from the a/w interface, thereby driving Marangoni flows that attract myelins growing from the C_12_E_3_ droplets. Furthermore, free‐floating C_12_E_3_ droplets can chase the OE‐CHO droplets along the channels they have created through the DA layer, featuring a predator–prey analogy. In contrast, at an a/w interface without a DA crystalline layer, the OE‐CHO droplet is rapidly attracted toward the C_12_E_3_ droplet, in analogy to strong drain droplets that we reported earlier^[^ ^42^ ^]^: Highlighting the unique role of the DA crystalline layer to drive the propulsion of OE‐CHO prey droplets and concomitantly mediate their interaction with C_12_E_3_ predators.

Finally, we foresee that the versatility of our design allows for extension with more complex molecular building blocks and interactions. Interesting feedback mechanisms may emerge from reactions that occur upon merging of predator and prey droplets, and for example enhance droplet motility, or stimulate regrowth of the DA crystalline layer to close off channels again. Nonreciprocal droplet–droplet interactions, and cascades of multidroplet actions and reactions – mediated by crystalline surfactant layers that provide dynamic barriers as structural control – open new pathways toward collectively operating droplet systems that self‐organize at air/water interfaces into reconfigurable, adaptive, and functional structures.

## Conflict of Interests

The authors declare no conflict of interest.

## Supporting information



Supporting Information

Supporting Information

Supporting Information

Supporting Information

Supporting Information

Supporting Information

Supporting Information

## Data Availability

The data that support the findings of this study are available in the supplementary material of this article.
