# Supplementary material for: Predator–Prey Behavior of Droplets Propelling Through Self‐Generated Channels in Crystalline Surfactant Layers
Source: Angew Chem Int Ed Engl. 2025 Apr 15;64(24):e202502352. doi: 10.1002/anie.202502352 (PMC12144871; doi:10.1002/anie.202502352)
Supplement: Supplementary file 1 — Supporting Information [file ANIE-64-e202502352-s004.pdf]

Predator-Prey Behavior of Droplets Propelling through Self-Generated Channels in Crystalline Surfactant Layers.

*Priyanshu Singh & Peter A. Korevaar**

Institute for Molecules and Materials, Radboud University, Heyendaalseweg 135, Nijmegen 6525 AJ, The Netherlands E-mail: p.korevaar@science.ru.nl

Supporting Information

Table of Contents

Materials 2

Methods 2

Supplementary Figures 6

Description of Supplementary Videos S1 – S6 16

Reference 16

Materials

The respective chemicals and materials were used as received: triethylene glycol monododecyl ether ($C_{12}E_3$, ≥ 95%) was purchased from TCI chemicals. Decylamine (≥ 95%), phenol (≥ 99%), oleic acid (≥ 99%), N,N-dicyclohexylcarbodiimide (DCC, ≥ 99%), dimethyl sulfoxide-D_6 (DMSO-D_6, ≥ 99.9%), dichloromethane (DCM, ≥ 99.8% anhydrous), celite and Oil red O dye were purchased from Sigma-Aldrich. 4-Hydroxy benzaldehyde (≥ 99%) was purchased from Acros Organics. 4-nitro phenol (≥ 99%), hydrochloric acid (2 M) and sodium hydroxide (2 M) were purchased from Fisher Scientific. Milli-Q water (18.2 MΩ.cm) was used throughout the experiments.

Methods

Aqueous bulk solution: An aqueous solution of 1.0 mM decylamine is used in all experiments, unless mentioned otherwise. All experiments were performed at room temperature.

Decylamine (DA) crystalline layer formation: To prepare the crystalline surfactant layer, we used an aqueous solution of 1 mM DA, and 0.5 µL DA was applied as a liquid droplet by a Gilson pipette, for experiments conducted in a 35 mm diameter Petri dish. For experiments conducted in a large Petri dish (100 mm diameter), 1.0 µL DA was applied.

OE-based droplet deposition: The OE-based droplets were deposited at the DA crystalline surfactant layer with a Gilson pipette.

Surface tension measurements: To measure the surface tension of the aqueous solutions, we used a force tensiometer (Biolin Scientific Sigma 701) with a platinum Wilhelmy plate (wetted length 39.2 mm). Before each measurement, the plate was rinsed with ethanol and heated with a flame torch until it became red hot. For every experiment, a 4.5 mL or 5 mL aqueous solution was added to a polystyrene Petri dish (Falcon, 35 mm) bottom part. In Figure S1, a 1.0 mM DA solution was used, and in Figure 5 a 0.5 mM $C_{12}E_3$ solution. The Wilhelmy plate was dipped at the air-water interface, such that the solution wetted the plate and formed a meniscus.

Optical microscopy: All optical microscopy images and videos were recorded with an Olympus IX73 dark-field inverted microscope equipped with a Point Grey Grasshopper3 camera. A 1.25x objective was used; brightness and condenser filters were adjusted appropriately, and the videos were recorded at 1 fps unless stated otherwise. We increased the contrast of the microscopy images to enhance the visibility of the DA layer, myelins and oil droplets by using ImageJ software in the Figures in the main text. In Fig. 2d, we used the polarized mode of the Olympus IX73 microscope. For this experiment, we used 2x and 4x magnification objectives and adjusted the condenser filter appropriately to observe the crystalline nature of the DA layer

OE-CHO oil droplet analysis by using a camera: For experiments shown in Figure 3d-e and Figure S8, we used a mirrorless camera (Nikon Z5) and macro lens (Laowa 100 mm f/2.8). The camera was mounted above the sample to capture the image. The sample was illuminated with a LED light panel (Viltrox VL-200T, colour temperature 5600 K) from the bottom and was diffused with a thin (3 mm) sheet of frosted polymethyl methacrylate. The camera settings were as follows, ISO: 100, shutter speed 1/40 s, aperture f22, capture rate: 0.5 fps.

Channel formation for directed myelin growth: We crafted 2 vertical pillars at 10 mm distance apart by placing a steel rod (thickness 1 mm, height 6 mm) in a Plexiglas plate (thickness 2 mm) upon heating the bottom part of the rod and then infusing it into the plate. The steel pillars allow to immobilize buoyant droplets at specific positions of the air-water interface. The concave meniscus formed by the pillars induces the “Cheerio’s” effect which attracts the droplet and keeps it in place. The Plexiglass plate with the steel pillars was placed in the Petri dish (lid of a Falcon 35 mm dish, diameter 38 mm, and height 4.5 mm, used as received) filled with the aqueous DA solution. The DA crystalline layer was

formed at the air-water interface by depositing a 0.5 µL DA droplet on top of a 1 mM DA solution in water. To create a channel amongst the pillars, we dipped the tip of 10 µL plastic pipette in the OE-CHO oil and moved it manually through the DA layer. Next, the $C_{12}E_3$ and OE-CHO droplets were deposited at the water-pillar contact point with a Gilson pipette.

Time-dependent positioning traces of drain droplets on microscopy: To obtain the time-dependent positioning traces of drain droplets deposited on the DA crystalline layer under the microscope, we used the manual tracking mode in ImageJ software. The (x, y) coordinates were obtained for each individual frame of the respective experiments and used to determine the velocity vs time of the drain droplets.

Time-dependent positioning traces of OE-CHO droplets: To obtain the time-dependent positioning traces acquired on camera recordings on the OE-CHO droplets deposited on the DA crystalline layer, we used the *imfindcircles* function in Matlab (R2022b) that was applied on contrast enhanced video frames. The (x,y) coordinates of the OE-CHO droplet were determined for each video frame, allowing to determine the droplet velocity as well. If the *imfindcircles* function failed to detect the circular appearance of the droplet for a particular frame, the (x,y) coordinates were recorded as *NaN*; leaving a blank spot in the plotted time-dependent positioning trace and the velocity vs time graph. The time-dependent positioning traces were overlayed on the camera recording that was contrast enhanced to improve the visibility of the DA crystalline layer with the channels formed by the OE-CHO droplets. For Figure 3d-e, the contrast was enhanced in Matlab (*imadjust* function), for Figure S8, the contrast was enhanced in ImageJ.

Synthesis of 4-formylphenyl oleate (OE-CHO): Following the procedure reported by Rostoll-Berenguer et al[1], oleic acid (12.25 g, 12 mmol) was loaded to a round bottom flask and dissolved in 10 mL DCM. The solution was kept at 0 °C, 4-hydroxybenzaldehyde (1.758 g, 15.6 mmol) was added and the solution was stirred for 5 min. N,N'-dicyclohexylcarbodiimide (DCC) (3.714 g, 18 mmol) was added, and the reaction mixture was kept stirring at 0 °C for another 10 min. Thereafter, the reaction mixture was kept at room temperature and stirred overnight. The crude reaction mixture was filtered over a pad of celite eluting with ethyl acetate to remove the dicyclohexylurea byproduct and unreacted reagents. The reaction mixture was washed with Milli-Q water 4 times (50 mL) in a separation funnel and dried over Mg_2SO_4. The solvent was evaporated, and the obtained crude product was purified by column chromatography with ethyl acetate/hexane (5:95 v/v) to yield OE-CHO.

^{1}H NMR (400 MHz, DMSO-D_6) δ (ppm): 10.0 (s, 1H), 7.98 (d, 2H), 7.36 (d, 2H), 5.33 (m, 2H), 2.62 (t, 2H), 1.99 (m, 4H), 1.66 (p, 2H), 1.48 – 1.29 (m, 20H), 0.85 (t, 3H).

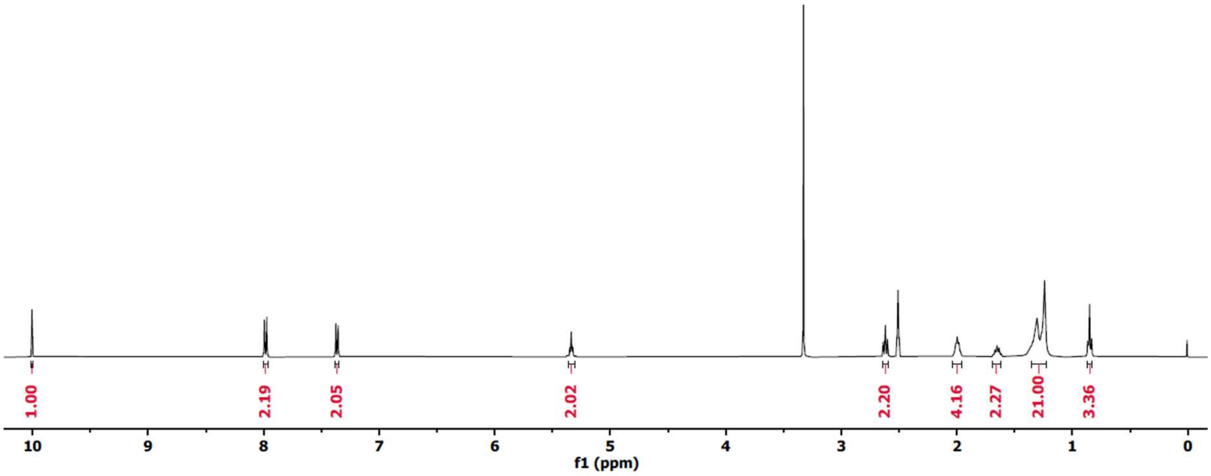

^{1}H NMR spectrum of OE-CHO recorded in DMSO-D_6

Synthesis of 4-nitrophenyl oleate (OE-NO_2): Oleic acid (1 g, 3.484 mmol) was loaded to a round bottom flask and dissolved in 10 mL DCM. The solution was kept at 0 °C, 4-nitrophenol (0.586 g, 4.1808 mmol, 1.2 eq.) was added and the solution was stirred for 5 min. N,N'-dicyclohexylcarbodiimide (DCC) (1.079 g, 5.226 mmol, 1.5 eq.) was added and the reaction mixture was kept stirring at 0 °C for another 10 min. Thereafter, the reaction mixture was kept at room temperature and stirred overnight. The crude reaction mixture was filtered over a pad of celite eluting with ethyl acetate to remove the dicyclohexylurea byproduct and unreacted reagents. The reaction mixture was washed with Milli-Q water (50 mL) in a separation funnel and dried over Mg_2SO_4. The solvent was evaporated, and the obtained crude product was purified by column chromatography with ethyl acetate/hexane (5:95 v/v) to yield OE-NO_2.

^{1}H NMR (400 MHz, DMSO-D_6) δ (ppm): 8.31 (d, 2H), 7.43 (d,2H), 5.34 (m, 2H), 2.64 (t, 2H), 1.99 (q, 4H), 1.65 (p, 2H), 1.31 (m, 20H), 0.85(t, 3H).

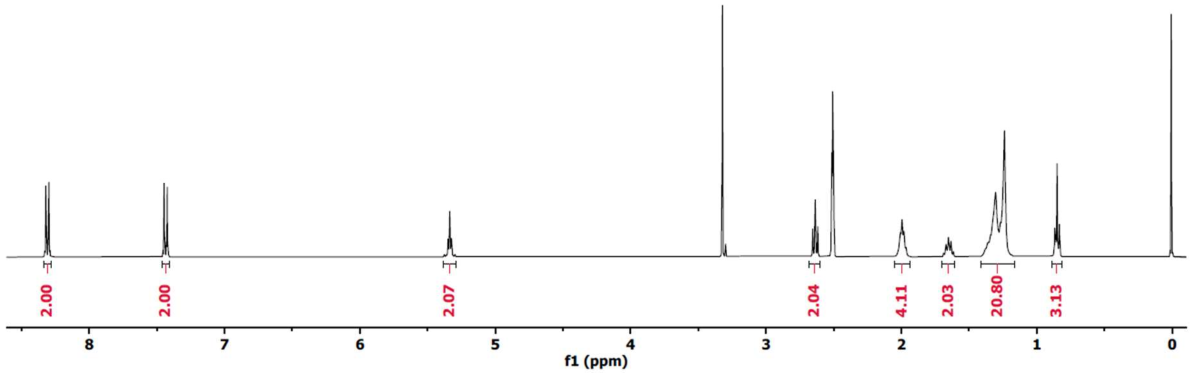

^{1}H NMR spectrum of OE-NO_2 recorded in DMSO-D_6

Synthesis of 4-methylphenyl oleate (OE-CH_3): Oleic acid (0.3 g, 1.05 mmol) was taken in a round bottom flask and dissolved in 10 mL DCM. The solution was kept at 0 °C, 4-methylphenol (0.137 g, 1.26 mmol, 1.2 eq.) was added and the solution was stirred for 5 min. N,N'-dicyclohexylcarbodiimide (DCC) (0.325 g, 1.575 mmol, 1.5 eq.) was added and the reaction mixture was kept stirring at 0 °C for another 10 min. Thereafter, the reaction mixture was kept at room temperature and stirred overnight. The crude reaction mixture was filtered over a pad of celite eluting with ethyl acetate to remove the dicyclohexylurea byproduct and unreacted reagents. The reaction mixture was washed with Milli-Q water 4 times (50 mL) in a separation funnel and dried over Mg_2SO_4. The solvent was evaporated, and the obtained crude product was purified by column chromatography with ethyl acetate/hexane (5:95 v/v) to yield OE-CH_3.

^{1}H NMR (400 MHz, $CDCl_3$) δ (ppm): 7.19 (d, 2H), 6.97 (d, 2H), 5.38 (m, 2H), 2.56 (t, 2H), 2.36 (s, 3H), 2.04 (q, 4H), 1.77 (p, 2H), 1.38 (m, 20H), 0.91 (t, 3H).

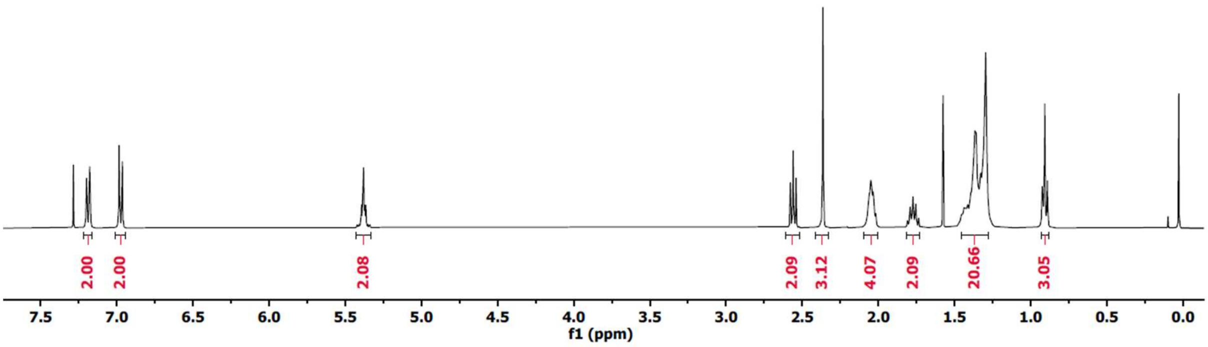

^{1}H NMR spectrum of OE-CH_3 recorded in $CDCl_3$

Synthesis of phenyl oleate (OE-H): Oleic acid (0.3 g, 1.05 mmol) was taken in a round bottom flask and dissolved in 10 mL DCM. The solution was kept at 0 °C, phenol (0.137 g, 1.26 mmol, 1.2 eq.) was added and the solution was stirred for 5 min. N,N'-dicyclohexylcarbodiimide (DCC) (0.325 g, 1.575 mmol, 1.5 eq.) was added and the reaction mixture was kept stirring at 0 °C for another 10 min. Thereafter, the reaction mixture was kept at room temperature and stirred overnight. The crude reaction mixture was filtered over a pad of celite eluting with ethyl acetate to remove the dicyclohexylurea byproduct and unreacted reagents. The reaction mixture was washed with Milli-Q water 4 times (50 mL) in a separation funnel and dried over Mg_2SO_4. The solvent was evaporated, and the obtained crude product was purified by column chromatography with ethyl acetate/hexane (5:95 v/v) to yield OE-H.

^{1}H NMR (400 MHz, $CHCl_3$) δ (ppm): 7.40 (d, 2H), 7.24 (m, 1H), 7.11(d, 2H), 5.38 (m, 2H), 2.58 (t, 2H), 2.05 (q, 4H), 1.78 (p, 2H), 1.30 (m, 20H), 0.91 (t, 3H)

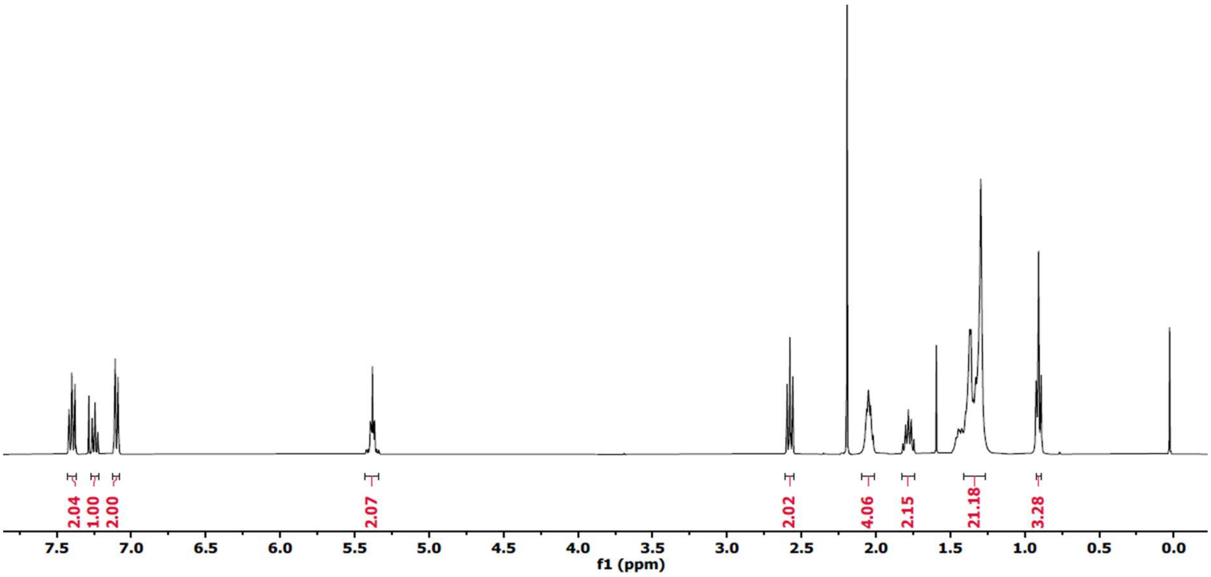

^{1}H NMR spectrum of OE-H recorded in $CDCl_3$

Supplementary Figures

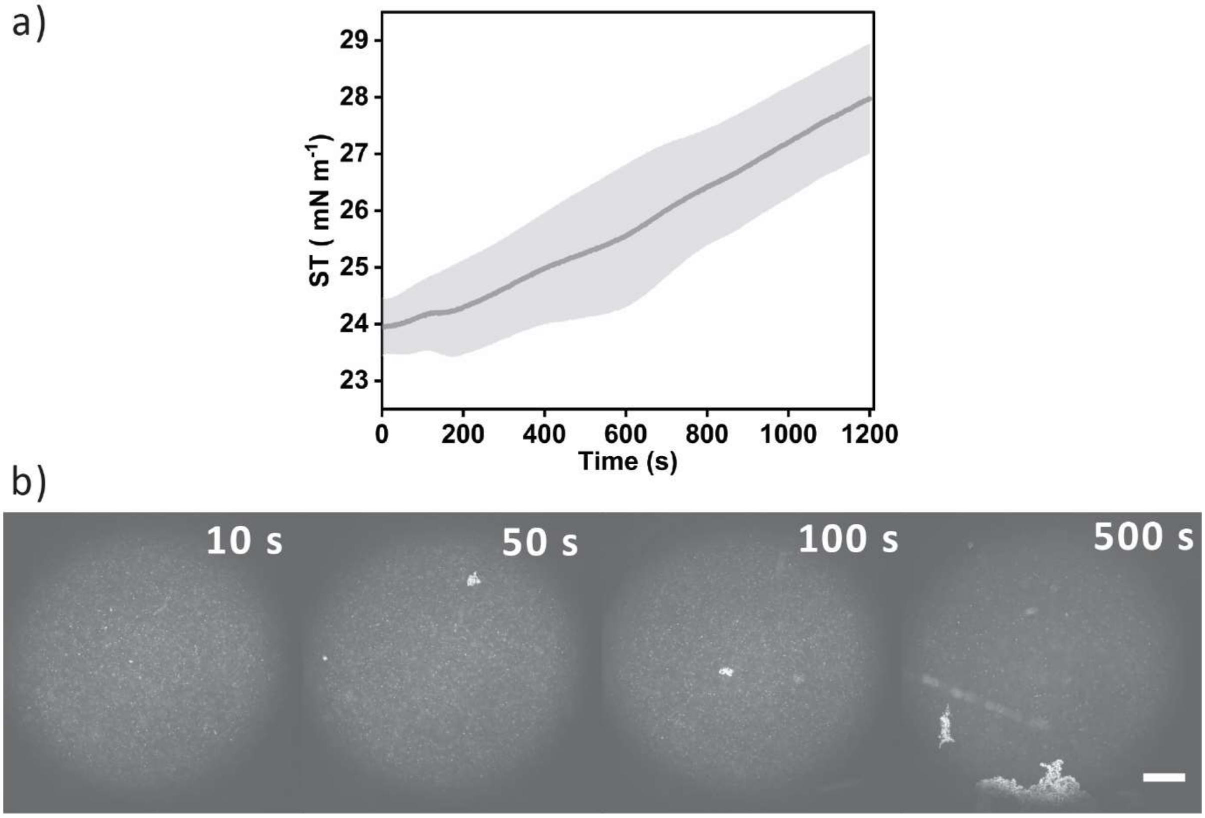

Figure S1: a) Surface tension (ST) measurements on an aqueous 1 mM DA solution (n = 3 experiments), using the Wilhelmy plate method. We ascribe the ST increase over time due to the aggregation of DA at the a/w interface. **b)** Optical microscopy recording of a 1 mM DA solution, that is freshly poured in a 3.5 cm Petri dish, showing the appearance of DA crystals floating at the a/w interface and accumulating at the edge of the Petri dish over time. In the first few minutes, no large DA crystals are observed at the a/w interface – indicating that the ST measurements at the onset of the experiment (t = [0 – 100 s]) in a) are not affected by DA crystallization. The scale bar represents 2 mm.

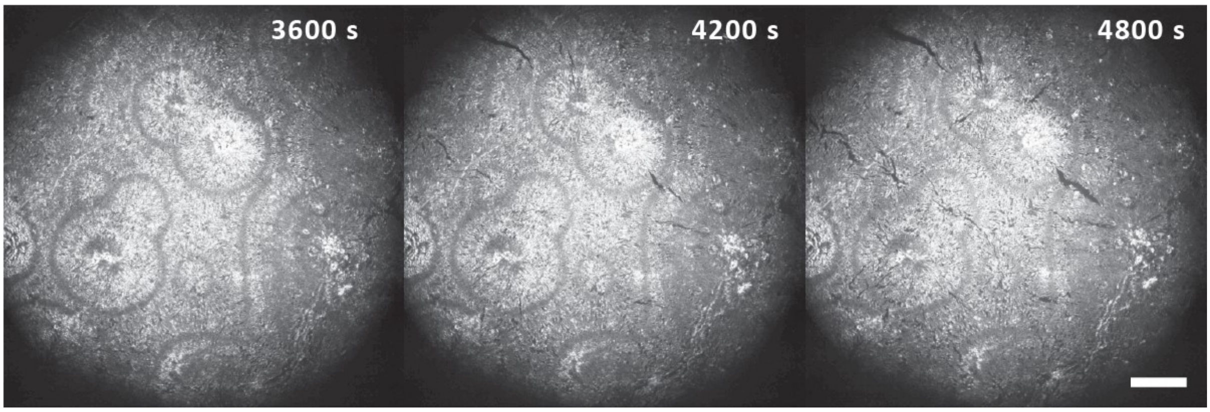

Figure S2: Optical microscopy recordings of decylamine (DA) crystalline layer. 1.0 µL DA was deposited on an aqueous solution of 1 mM DA. The crystalline layer is stable for up to 3600 s, but later cracks start to appear. The scale bar represents 2 mm.

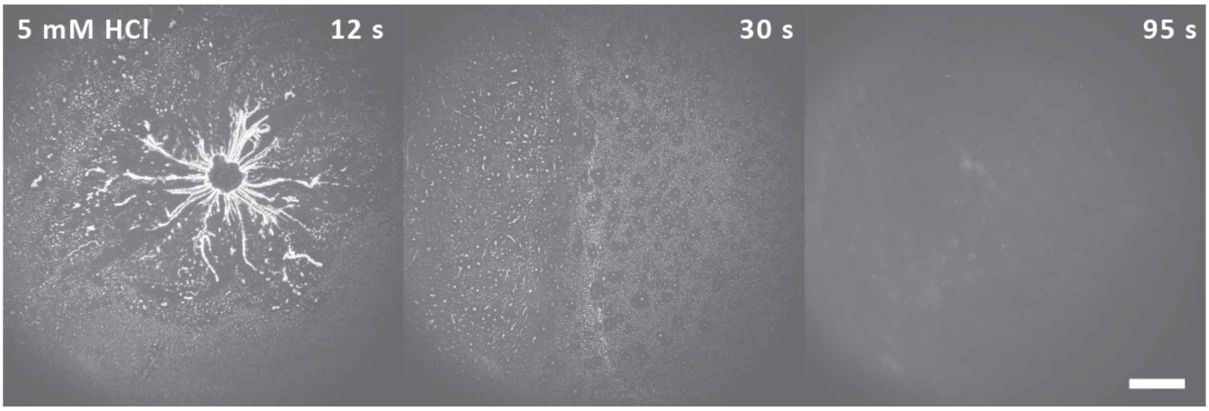

Figure S3: Optical microscopy recordings of a DA droplet (1.0 µL) deposited on an aqueous 5 mM HCl solution. The crystalline layer does not form and the DA droplet is observed to dissolve in the solution over the course of approx. 30 seconds, resulting in a clear solution as exemplified by the image recorded at t = 95 s. The scale bar represents 2 mm.

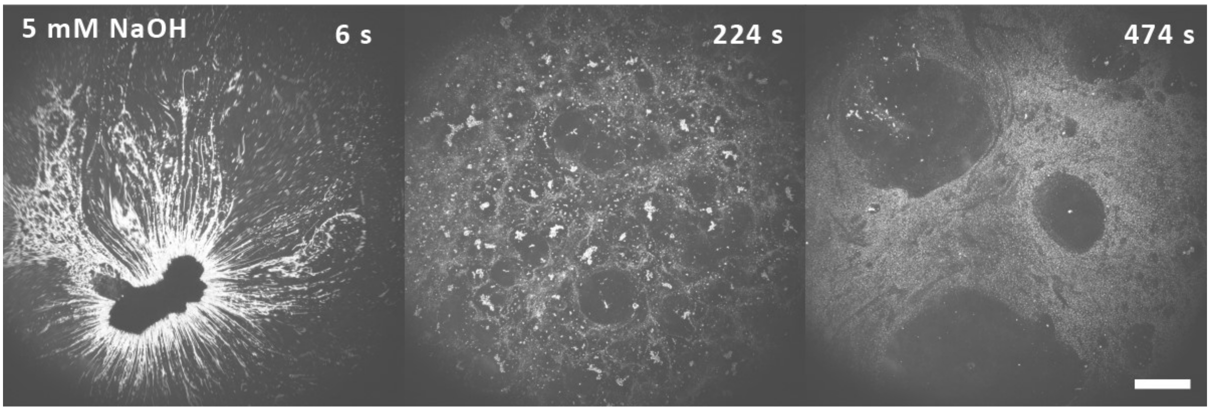

Figure S4: Optical microscopy recordings of a DA droplet (1.0 µL) deposited on an aqueous 5 mM NaOH solution. The crystalline layer does not form. Instead, a wave-pattern of phase-separated microdroplets is observed at the air/water interface. The scale bar represents 2 mm.

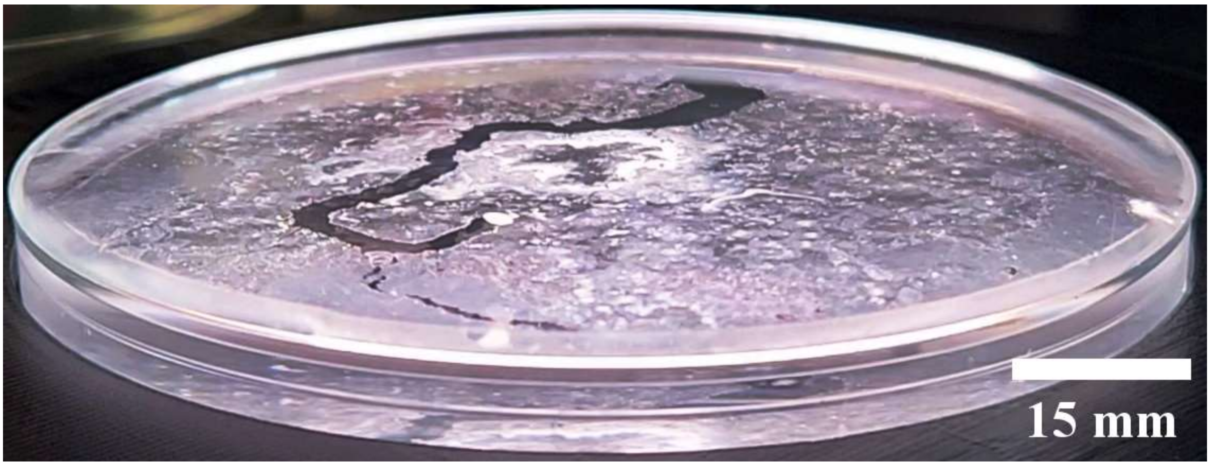

Figure S5: Upon depositing onto the DA layer, the OE-CHO droplet shows a self-propelled motion and creates a millimeter-wide channel through the DA layer. The photograph of the Petri dish is acquired under the same angle as in Fig. 2c.

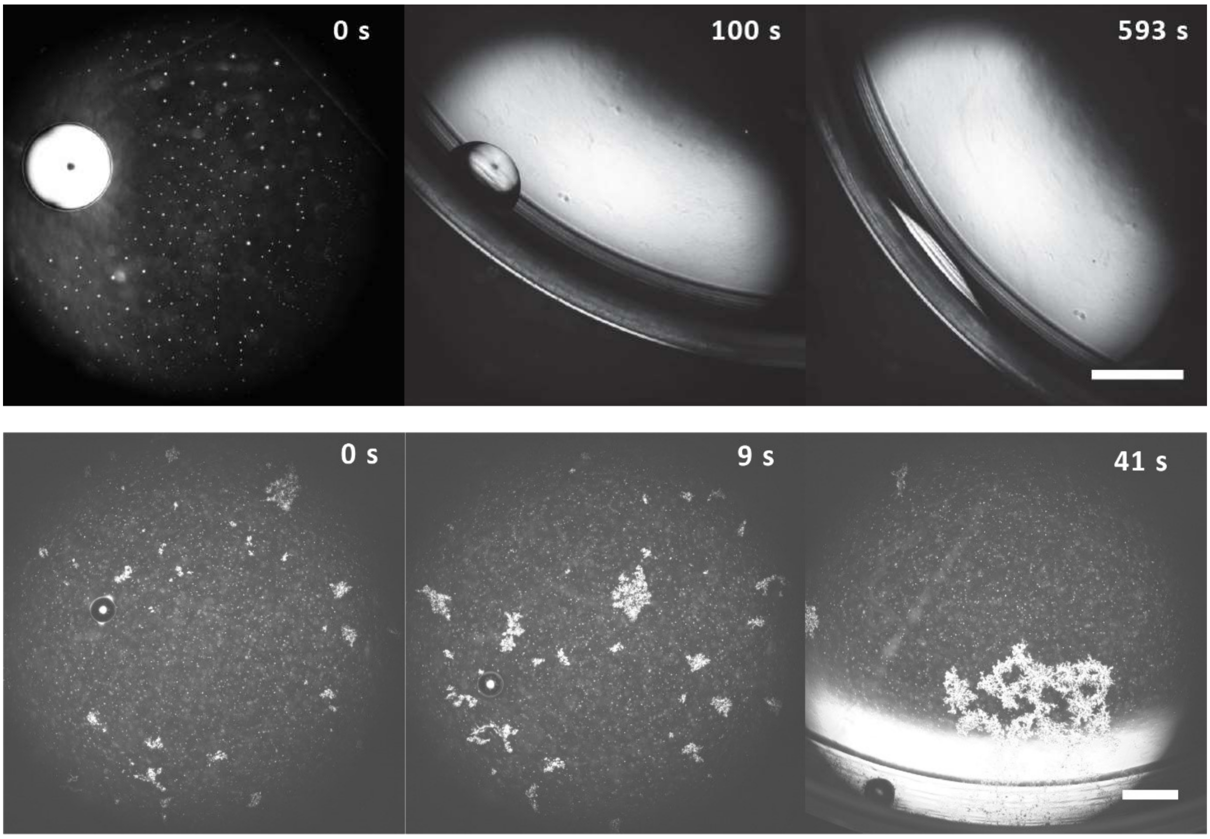

Figure S6: Top row: Optical microscopy recordings of an OE-CHO droplet (1.0 µL) deposited on MQ water. The droplet was deposited in the middle of the Petri dish (35 mm diameter), and was observed to bounce rapidly to the wall of the dish, and wet the wall of the dish at t = 593 s. The magnification is 2x and the scale bar represents 2 mm. **Bottom row:** Optical microscopy recordings of an OE-CHO droplet (0.5 µL) deposited on a 1 mM DA solution. The droplet was deposited in the middle of the Petri dish (35 mm diameter) and was observed to attract DA crystals floating at the a/w interface – indicative of a Marangoni flow directed towards the droplet that is driven by depletion of DA surfactants from the a/w interface. Next, the droplet was observed to bounce rapidly to the wall of the dish, and wet the wall of the dish at t = 41 s. The magnification is 1.25x and the scale bar represents 2 mm.

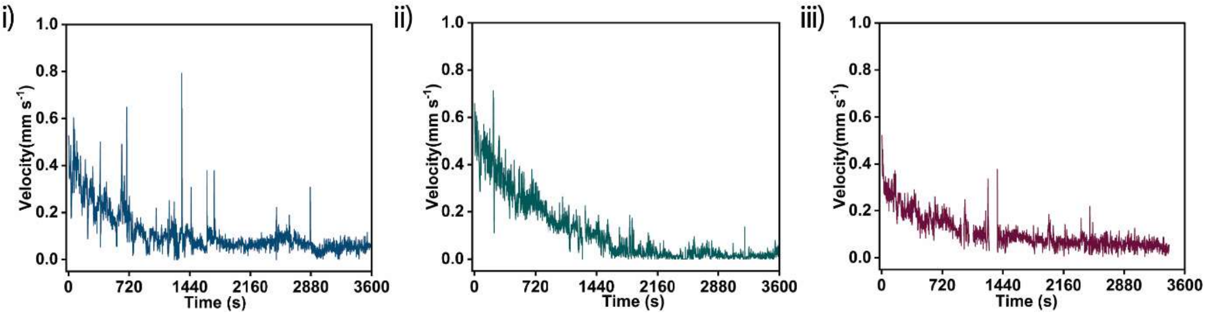

Figure S7: Velocity vs time graphs acquired on 3 replicate experiments of an OE-CHO droplet (1.0 µL) self-propelling through a DA crystalline layer on top of an aqueous DA solution (1 mM, 60 mL) in a 100 mm x 15 mm Petri dish. The graphs correspond to the experiments shown in Fig. 3d (i), Fig. 3e left (ii) and Fig. 3e right (iii), respectively.

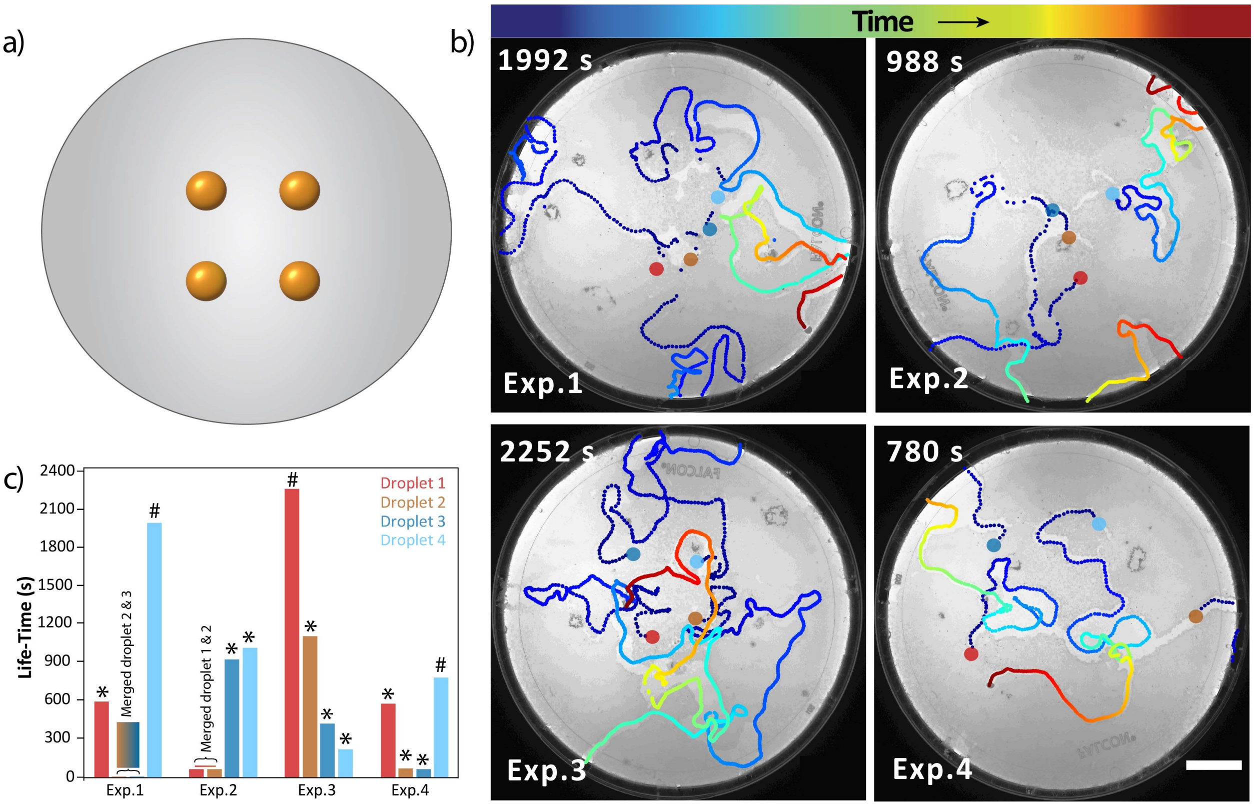

Figure S8. Concomitant self-propelled motion of 4 OE-CHO oil droplets through the DA crystalline layer. **a)** At the start of the experiment, four 1.0 μL OE-CHO droplets are deposited on the DA crystalline layer (100 mm diameter Petri dish). **b)** Time-dependent positioning traces acquired on camera recordings of four replicate experiments, where the paths of the individual OE-CHO droplets are tracked over time. The displayed images are acquired at the end of the respective experiments, when all droplets have stopped moving. The scale bar represents 2 mm. **c)** Lifetime of individual droplets in the four replicate experiments shown in b). Merge of droplets is indicated with the accolade in exp. 1 and 2. Droplets indicated with * have bounced to wall of the Petri dish (ending their lifetime); Droplets indicated with # have stopped moving while embedded in the DA crystalline layer.

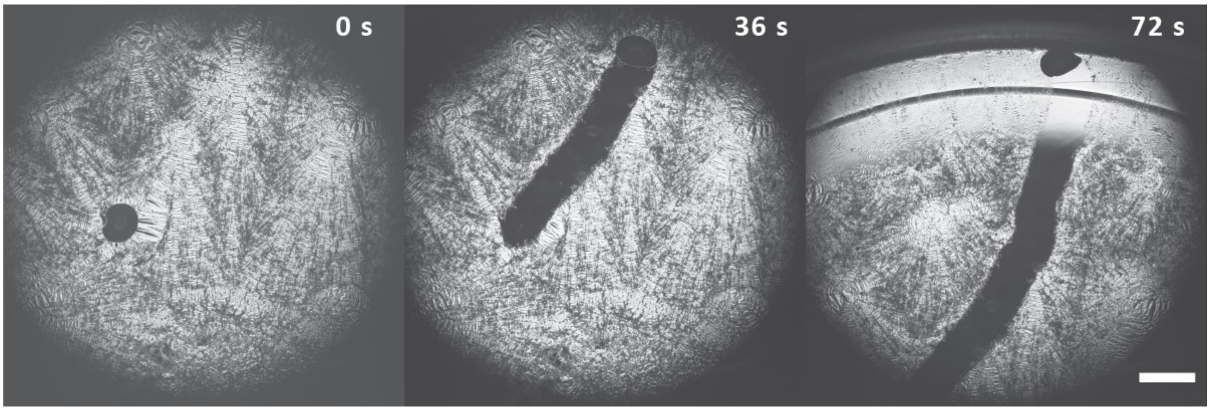

Figure S9: Optical microscopy recordings of an OE-CHO droplet (0.5 μL) loaded with 20 v/v% DA, deposited on a DA crystalline layer formed on an aqueous DA solution (1 mM) in a Petri dish (35 mm diameter). The scale bar represents 2 mm.

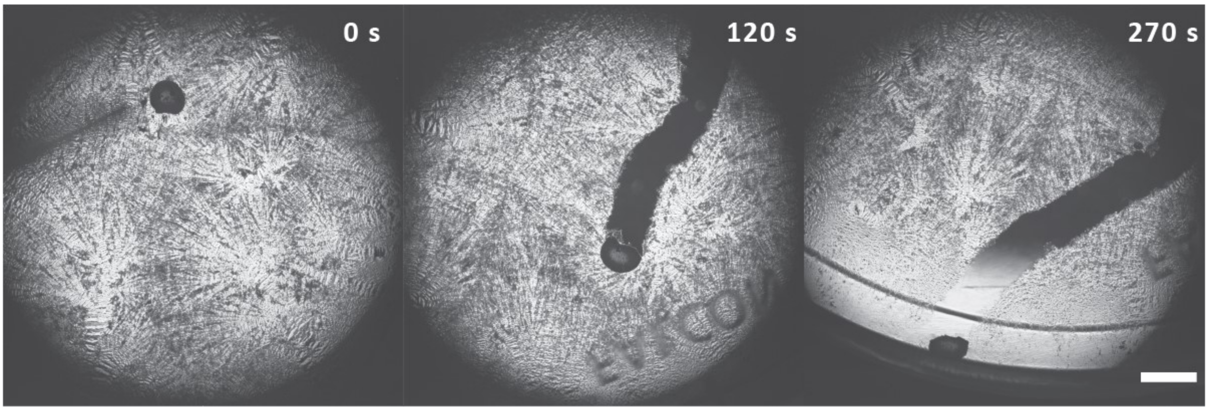

Figure S10: Optical microscopy recordings of an OE-CHO droplet (0.5 µL) loaded with 25 v/v% DA, deposited on a DA crystalline layer formed on an aqueous DA solution (1 mM) in a Petri dish (35 mm diameter). The scale bar represents 2 mm.

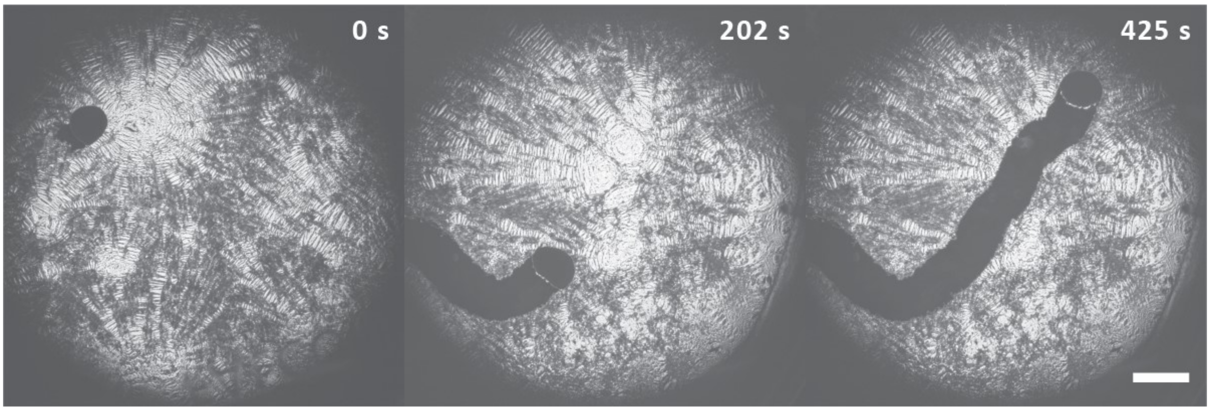

Figure S11: Optical microscopy recordings of an OE-CHO droplet (0.5 µL) loaded with 30 v/v% DA, deposited on a DA crystalline layer formed on an aqueous DA solution (1 mM) in a Petri dish (35 mm diameter). The scale bar represents 2 mm.

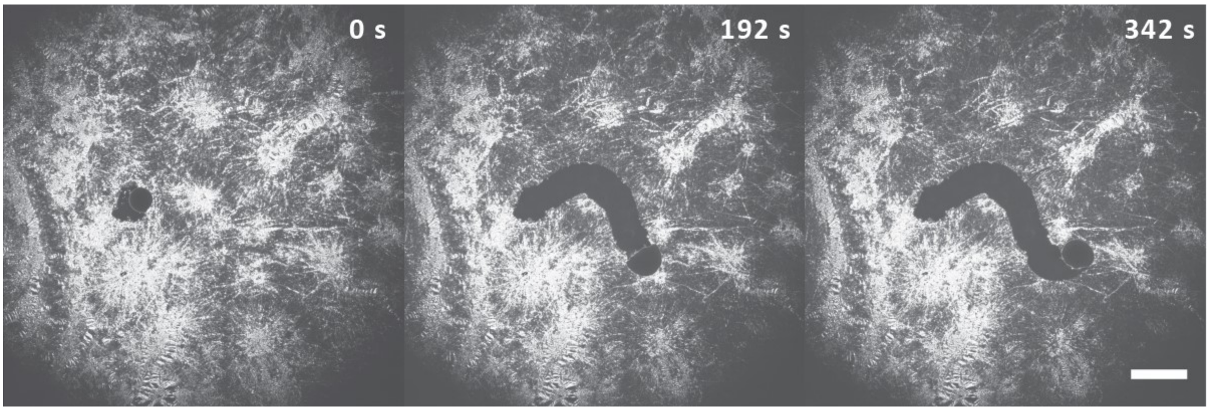

Figure S12: Optical microscopy recordings of an OE-CHO droplet (0.5 µL) loaded with 35 v/v% DA, deposited on a DA crystalline layer formed on an aqueous DA solution (1 mM) in a Petri dish (35 mm diameter). The scale bar represents 2 mm.

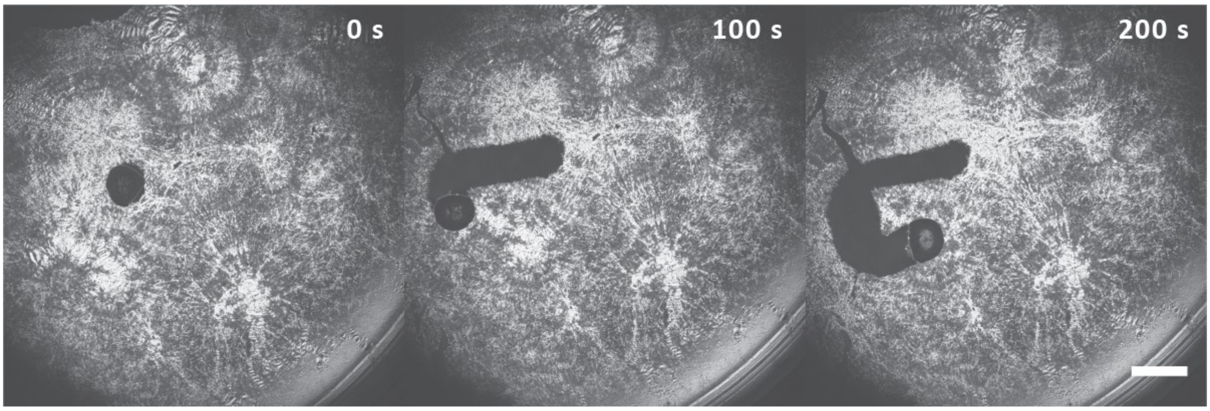

Figure S13: Optical microscopy recordings of an OE-CHO droplet (0.5 µL) loaded with 36 v/v% DA, deposited on a DA crystalline layer formed on an aqueous DA solution (1 mM) in a Petri dish (35 mm diameter). The scale bar represents 2 mm.

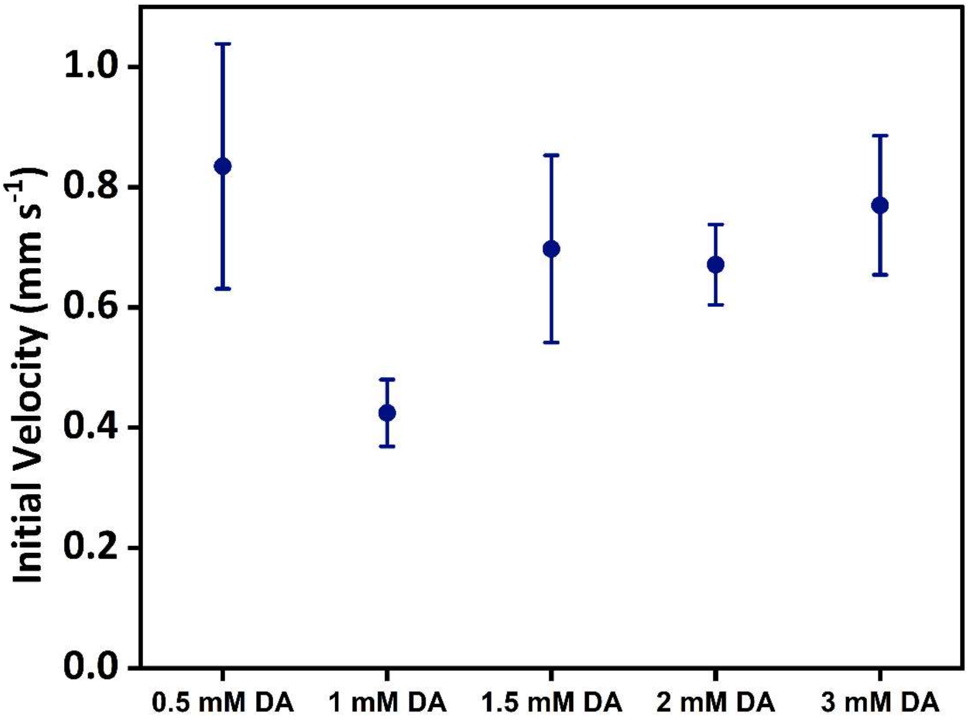

Figure S14: Graph of the initial velocity of OE-CHO droplets (0.5 µL) through a DA crystalline layer vs DA concentration in the underlying aqueous solution. The velocity is acquired over the first 18 s after deposition, n = 3 separate experiments per DA concentration.

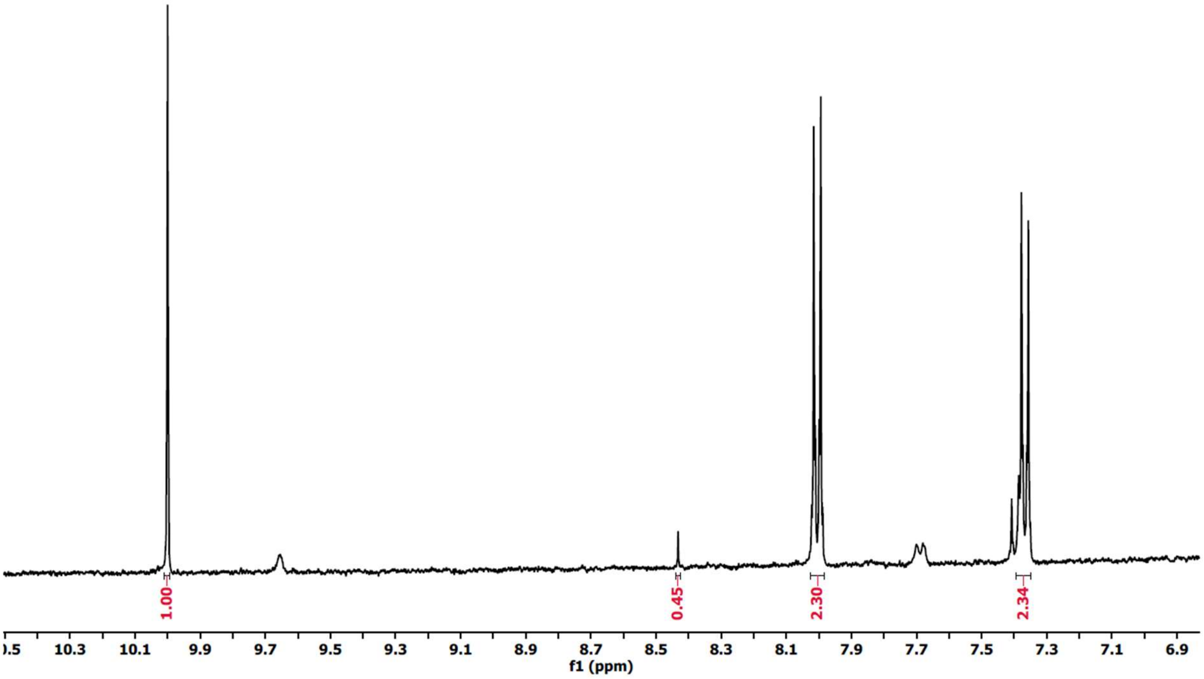

Figure S15: ^{1}H NMR spectrum acquired on a 15 µL OE-CHO droplet that has been in contact with a DA layer (15 µL droplet) formed on an aqueous 1 mM DA solution (5.5 mL in 35 mm Petri dish) for a period of 30 min, and subsequently extracted with a Gilson pipetted and dispersed in DMSO-D_6. The peak corresponding to the –CHO proton of the aldehyde (10.0 ppm) has a relative integral of 1.00; the peak corresponding to the –CH=N– proton of the imine (8.45 ppm) has an integral of 0.45. These values indicate that the extracted OE-CHO droplet contained approx. 31 mol% imine.

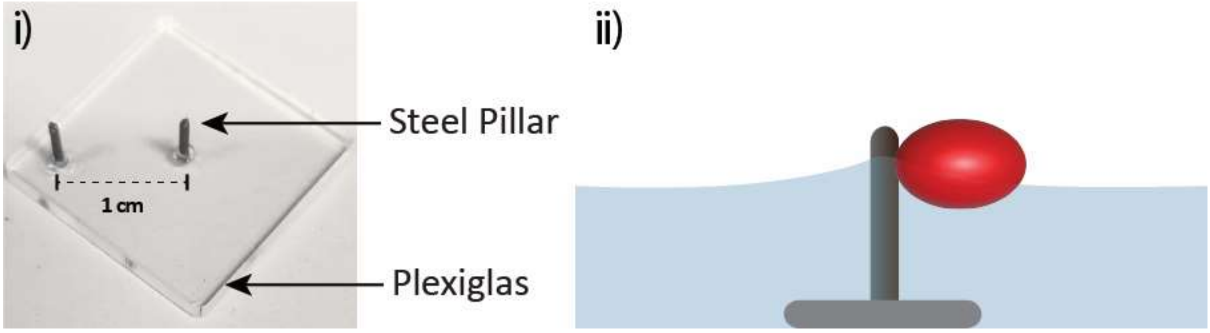

Figure S16: i) Photograph of steel pillars mounted in the Plexiglas base plate 1 cm apart. ii) Schematic representation of the pillar in solution, with positive meniscus. The "Cheerio's" effect causes the droplets to stick to the pillar and keep them in place. This setup is used in the experiments shown in Figure 5 of the main text.

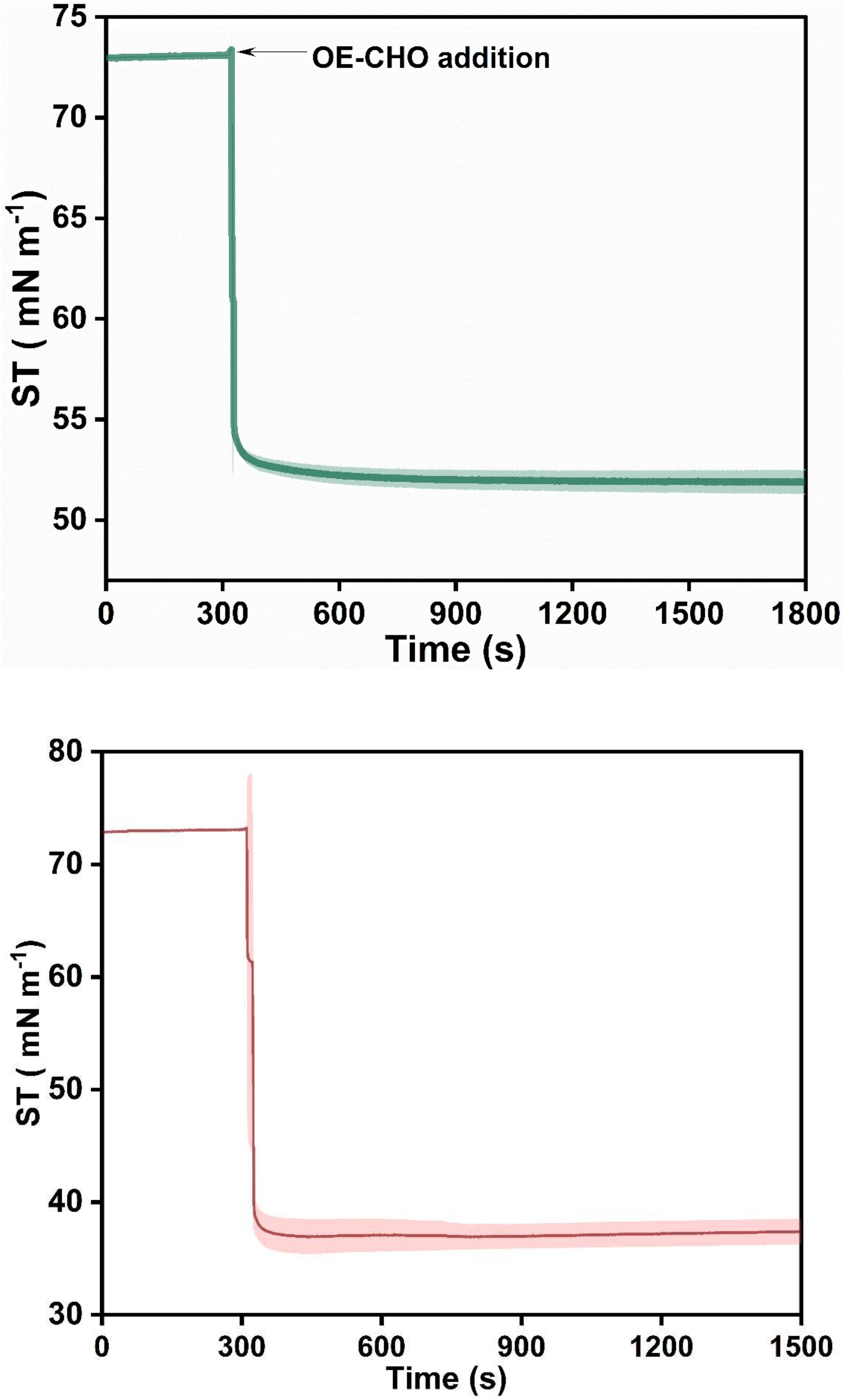

Figure S17: Surface tension (ST) measurements on MQ water, upon deposition of an OE-CHO droplet (0.5 µL) at the a/w interface (**top**), and (**bottom**) upon deposition of an OE-CHO droplet with 20 v/v% DA (0.5 µL) at the a/w interface. Both graphs are based on n = 3 separate experiments. During the measurements, the droplets were deposited at approx. 300 s.

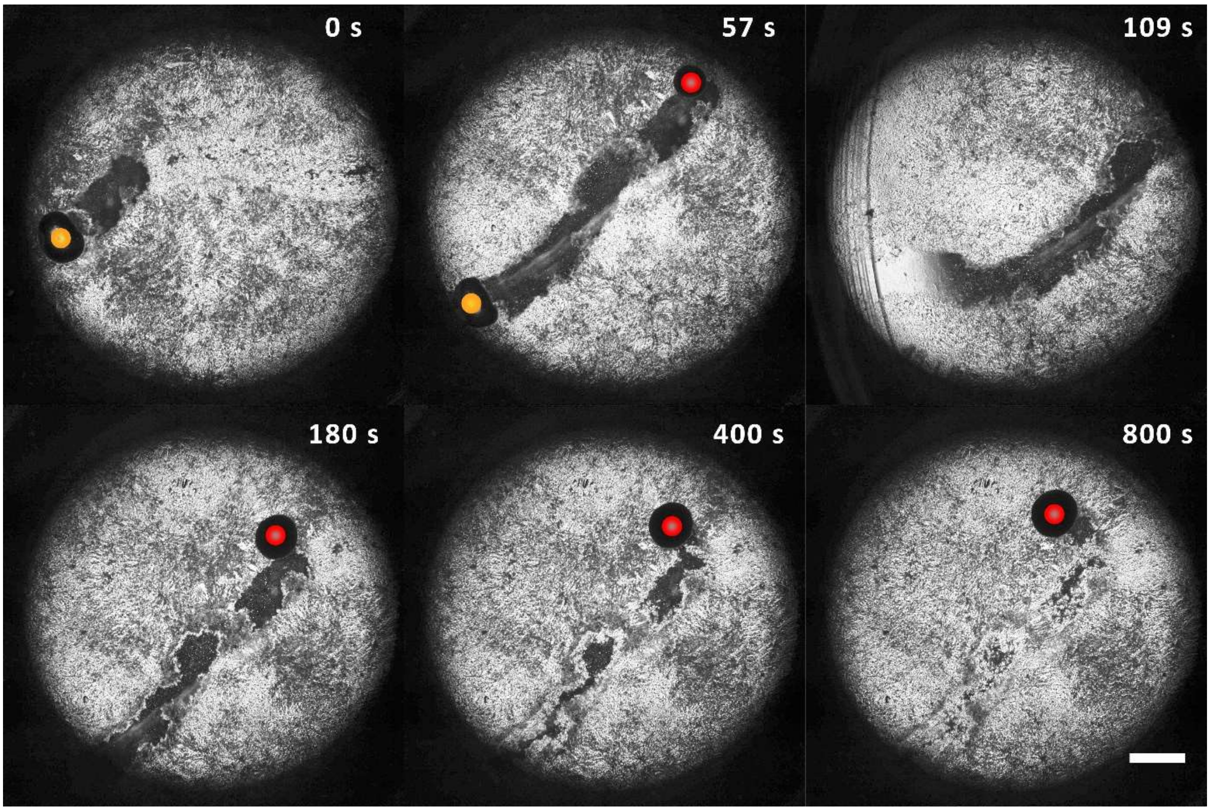

Figure S18: Optical microscopy recordings of an OE-CHO droplet (1.0 µL), deposited on a DA crystalline layer that has spontaneously formed at the a/w interface of a 2.5 mM DA aqueous solution in a Petri Dish (35 mm diameter). The OE-CHO droplet (indicated by yellow sphere) moves through the crystalline layer, creates an open channel and bounces to the wall of the Petri dish at t = 109 s. At t = 57 s, a $C_{12}E_3$ droplet (1.0 µL, red sphere) was deposited in the open channel but no myelin growth was observed over time. From approx. t = 180 s, the DA crystalline layer was observed to regenerate within the open channel that was created by the OE-CHO droplet, resulting in a completely renewed crystalline layer closing the channel around t = 800 s. The scale bar represents 2 mm.

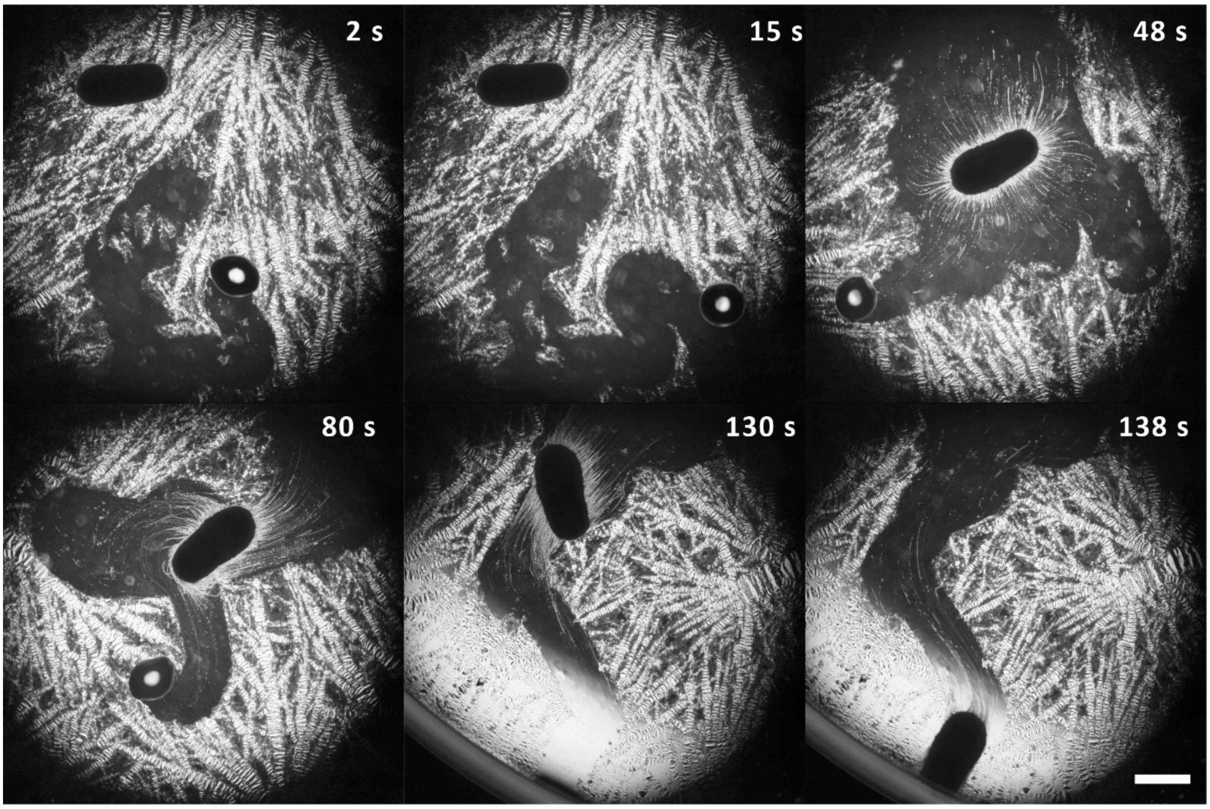

Figure S19: Optical microscopy recordings showing the predator-prey interaction of 1 $C_{12}E_3$ droplet (0.5 µL) and 1 OE-CHO droplet (0.5 µL), deposited on a DA crystalline layer formed on 5.5 mL of a 0.5 mM DA aqueous solution. The $C_{12}E_3$ droplet started chasing the OE-CHO droplet at t = 48 s. The OE-CHO droplet bounced to the wall of the Petri dish (35 mm diameter), and the $C_{12}E_3$ droplet caught the OE-CHO droplet at t = 138 s. The scale bar represents 2 mm.

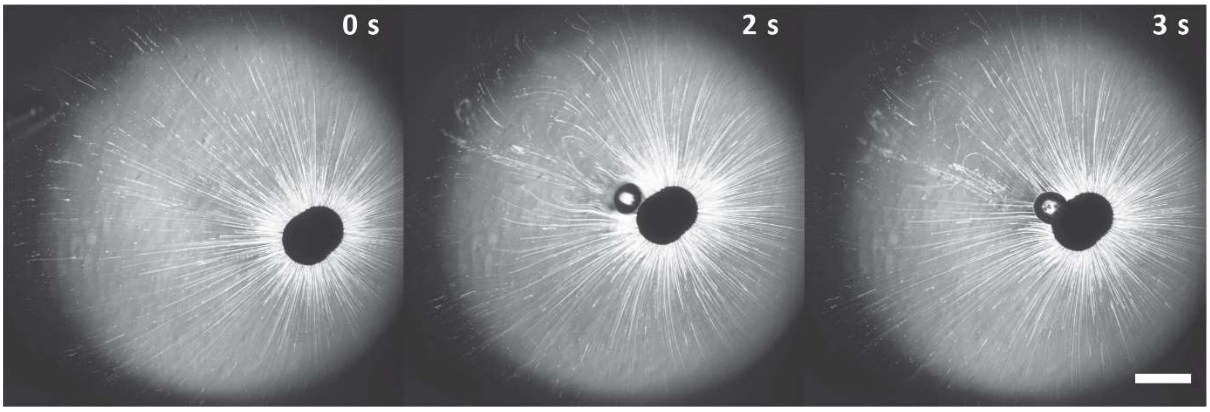

Figure S20: Optical microscopy recording showing a $C_{12}E_3$ (1.0 µL) and OE-CHO (1.0 µL) droplet deposited at the a/w interface of Milli-Q water (5.5 mL) in a 35 mm Petri dish. The OE-CHO droplet attracts myelins, moves closer to the $C_{12}E_3$ droplet (t = 2 s) and merges with the $C_{12}E_3$ droplet (t = 3 s). The image at t = 0 s shows the system right before the deposition of the OE-CHO droplet. The scale bar represents 2 mm.

Description of Supplementary Videos S1 – S6

File Name: Video S1 – Optical microscopy recording corresponding to Figure 2b. Decylamine droplet (0.5 µL) deposited on a 1 mM DA aqueous solution at the air/water interface. The DA droplet forms short transient filaments, which are simultaneously released to form a crystalline layer at the a/w interface.

File name: Video S2 – Optical microscopy recording corresponding to Figure 3b-c. The self-propelled motion of a OE-CHO (0.5 µL) oil droplet on a DA crystalline layer. The OE-CHO consumes the DA crystalline layer, creating a millimeter-wide open channel and the shape of OE-CHO changes during the motion. The oil droplet avoids open channels by changing its direction of motion and showing a self-evading behavior.

File name: Video S3 – Camera recordings corresponding to Figure 3d-e. Three replicate experiments on the self-propelled motion of an OE-CHO oil droplet (1.0 µL) through a DA crystalline layer formed on a large size Petri dish (100 mm x 15 mm). The self-propelled motion was sustained for 60 min. The movie is 30x sped up.

File name: Video S4 – Camera recordings corresponding to Figure S8. Four replicate experiments on the self-propelled motion of 4 OE-CHO oil droplets (1.0 µL) through a DA crystalline layer formed on a large size Petri dish (100 mm x 15 mm). The OE-CHO droplets merged, bounced to the wall of the Petri dish or stopped moving after a certain time; showing the variability in the behavior of OE-CHO droplets. The movie is 30x sped up.

File name: Video S5 – Optical microscopy recording corresponding to Figure 5e-f. A $C_{12}E_3$ droplet (1.0 µL) is positioned at one end (top) of a 1 cm long open channel formed in a DA crystalline layer. The myelins started to grow from the $C_{12}E_3$ droplet after introducing the OE-CHO oil droplet (0.5 µL) at the other end of the channel (bottom). **Left movie:** OE-CHO droplet; **middle movie:** OE-CHO droplet with 15 v/v% DA; **right movie:** OE-CHO droplet with 20 v/v% DA.

File name: Video S6 – Optical microscopy recording corresponds to Figure 6. Three replicate experiments on the predator-prey interaction of 4 $C_{12}E_3$ droplets (0.5 µL) with a OE-CHO oil droplet (0.5 µL) deposited on the DA crystalline layer at the air-water interface. **Top row:** OE-CHO droplet; **middle row:** OE-CHO droplet with 15 v/v% DA; **bottom row:** OE-CHO droplet with 20 v/v% DA.

The timers in the Videos indicate mm:ss:hundreds of seconds.

Reference

[1] J. Rostoll-Berenguer, G. Blay, J. R. Pedro, C. Vila, *Organic Letters* **2020**, *22*, 8012–8017.
